# Aspirin Use and Common Cancer Risk: A Meta-Analysis of Cohort Studies and Randomized Controlled Trials

**DOI:** 10.3389/fonc.2021.690219

**Published:** 2021-06-18

**Authors:** Lijuan Wang, Rongqi Zhang, Lili Yu, Jiarui Xiao, Xuan Zhou, Xinxuan Li, Peige Song, Xue Li

**Affiliations:** ^1^ School of Public Health and the Second Affiliated Hospital, Zhejiang University School of Medicine, Hangzhou, China; ^2^ Centre for Global Health, Usher Institute, University of Edinburgh, Edinburgh, United Kingdom

**Keywords:** aspirin, cancer, randomized controlled trial, cohort study, meta-analysis

## Abstract

**Background:**

Whether aspirin use can decrease or increase cancer risk remains controversial. In this study, a meta-analysis of cohort studies and randomized controlled trials (RCTs) were conducted to evaluate the effect of aspirin use on common cancer risk.

**Method:**

Medline and Embase databases were searched to identify relevant studies. Meta-analyses of cohort studies and RCTs were performed to assess the effect of aspirin use on the risk of colorectal, gastric, breast, prostate and lung cancer. Cochran Q test and the I square metric were calculated to detect potential heterogeneity among studies. Subgroup meta-analyses according to exposure categories (frequency and duration) and timing of aspirin use (whether aspirin was used before and after cancer diagnosis) were also performed. A dose-response analysis was carried out to evaluate and quantify the association between aspirin dose and cancer risk.

**Results:**

A total of 88 cohort studies and seven RCTs were included in the final analysis. Meta-analyses of cohort studies revealed that regular aspirin use reduced the risk of colorectal cancer (CRC) (RR=0.85, 95%CI: 0.78-0.92), gastric cancer (RR=0.67, 95%CI: 0.52-0.87), breast cancer (RR=0.93, 95%CI: 0.87-0.99) and prostate cancer (RR=0.92, 95%CI: 0.86-0.98), but showed no association with lung cancer risk. Additionally, meta-analyses of RCTs showed that aspirin use had a protective effect on CRC risk (OR=0.74, 95%CI: 0.56-0.97). When combining evidence from meta-analyses of cohorts and RCTs, consistent evidence was found for the protective effect of aspirin use on CRC risk. Subgroup analysis showed that high frequency aspirin use was associated with increased lung cancer risk (RR=1.05, 95%CI: 1.01-1.09). Dose-response analysis revealed that high-dose aspirin use may increase prostate cancer risk.

**Conclusions:**

This study provides evidence for low-dose aspirin use for the prevention of CRC, but not other common cancers. High frequency or high dose use of aspirin should be prescribed with caution because of their associations with increased lung and prostate cancer risk, respectively. Further studies are warranted to validate these findings and to find the minimum effective dose required for cancer prevention.

## Introduction

Cancer is one of the leading causes of death around the world, with approximately one in six deaths resulting from cancers (1). Globally, it is estimated that there were around 19.3 million new cancer cases and 10 million cancer deaths in 2020 (2). The most common cancers that occur in men or women include breast, lung, colorectal, gastric and prostate cancer, contributing about 50% of the total number of new cases diagnosed each year (3). It is estimated that about 30-50% of cancers can be prevented by avoiding risk factors and implementing existing evidence-based prevention strategies (4, 5). Primary prevention of cancer has been an important public health issue and the use of drugs for chemoprevention is of particular importance (6).

Aspirin (acetylsalicylic acid) is one of the most commonly used drugs in primary and secondary prevention of cardiovascular diseases (CVDs). Recently, the possible anti-cancer effect of aspirin has gained much attention, with extensive research efforts focusing on elaborating its effectiveness in the prevention of colorectal cancer (CRC), gastric cancer, breast cancer, prostate cancer and lung cancer (7–9). In analyses that included six trials of daily low-dose aspirin in primary prevention, aspirin treatment was found to be associated with an approximately 20% reduction in overall cancer incidence between 3 years and 5 years after initiation of the intervention and a 30% reduction during follow up >5 years. In analyses that included 34 trials of daily aspirin at various doses, cancer mortality was also found to have reduced during the >5 years of follow up (10). By far, the chemopreventive effect of aspirin has been convincingly established for CRC. Early in 2016, the U.S. Preventive Services Task Force (USPSTF) has issued a clinical recommendation that a routine use of low-dose aspirin for the primary prevention of CVDs in the elderly is likely to yield substantial additional benefits with regard to CRC prevention, reflecting the accumulating evidence for a chemopreventive effect of low-dose aspirin against cancer (11).

Aspirin is distinguished as a promising pharmacologic agent for chemoprevention of cancer. New research has reinforced the idea that long-term low-dose aspirin intake may inhibit cancer cell proliferation and metastasis (12, 13). Findings from observational studies continue to hint at the anti-cancer potential of aspirin against a variety of cancers (14–17). The USPSTF findings emphasized the need for more research efforts in evaluating the preventive effects of aspirin on different cancer sites. The associations between aspirin intake and the risk of a wide range of cancers (e.g., gastric, breast, prostate and lung cancer) have been observed in epidemiological studies, however, given the existence of substantial heterogeneity among studies, the evidence is less consistent and could have been subject to multiple forms of bias (18). Assessing evidence from every possible source is therefore needed before the role of aspirin in clinical practice can be more clearly defined.

This meta-analysis included cohort studies and randomised controlled trials (RCTs) that assessed the effects of aspirin on common cancers. By exploring the relationship between aspirin use and common cancer risk, we aimed to provide evidence for cancer-related implications of aspirin use. The study will not only inform patient-physician decision-making about the optimal use of aspirin, but also be of significant value to the research community.

## Materials and Methods

### Literature Search

Medline and EMBASE databases were systematically searched from inception to 16 October 2020 by using a comprehensive search strategy (**Supplementary Table 1**) to identify relevant studies. Mesh Terms and key words used for literature search included “(Aspirin OR Aspirins OR Acetyl Salicylic Acid OR ASA OR Acetylsalicylic Acid OR Acetylsalicylic OR acetylsalicylate OR salicylic acid OR salicylate OR 2-(Acetyloxy)benzoic Acid OR 2-Acetoxybenzoic Acid OR o-Acetylsalicylic Acid OR o-Acetoxybenzoic Acid OR Acylpyrin OR Aloxiprimum OR Colfarit OR Dispril OR Easprin OR Ecotrin OR Endosprin OR Magnecyl OR Micristin OR Polopirin OR Polopiryna OR Solprin OR Solupsan OR Zorprin OR Acetysal) AND (cancer OR cancers OR neoplasm OR neoplasms). All identified records went through a three-step parallel review of title, abstract and full text based on pre-defined inclusion and exclusion criteria. Two investigators (L.W., R.Z.) conducted literature searches, assessed the eligibility of retrieved publications independently. In case of any discrepancy, the final decision was made after discussion.

### Inclusion and Exclusion Criteria

This study included cohort studies and RCTs (intervention with aspirin intake vs. placebo or no treatment) that examined associations between aspirin use and common cancer outcomes (i.e., colorectal, gastric, breast, prostate and lung cancer). Outcomes of interest included cancer incidence and mortality. When multiple reports were published based on the same study, either the most recent one with the longest period of follow-up or the one with the most comprehensive data was included. We excluded (i) studies that investigated associations between aspirin and non-cancer outcomes; (ii) studies that evaluated non-oral forms or derivatives of aspirin; (iii) RCTs that included interventions of non-aspirin antithrombotic medications (e.g., warfarin), or aspirin treatment in combination with other nonsteroidal anti-inflammatory drugs (e.g., naproxen, ibuprofen) or a chemo-preventive agent (e.g., tamoxifen) and (iv) conference abstracts, reviews, comments, animal and molecular studies.

### Data Extraction

For each eligible study, data were extracted on first author, year of publication, study population and settings, cancer outcomes, the number of events and sample size, aspirin use categories according to frequency (e.g., regular or daily) and duration (e.g., ≥ 5 years), aspirin dose and corresponding maximally adjusted relative risk (RR) or hazard ratio (HR) or standardized incidence/mortality ratio (SIR/SMR) with 95% confidence intervals (CIs). Data extraction was performed by one investigator (L.W.) and verified by another two investigators (L.Y. and R.Z.).

### Statistical Analysis

Relative risk with 95% CI was considered the common measurement of the associations between aspirin use and cancer risk. Because the absolute risk of cancer is low, it was assumed that HRs and SIRs/SMRs were similar to RRs. Heterogeneity among studies was first detected using Cochran Q test and the I square metric (19). If studies were significantly heterogeneous (*P*<0.10 and I^2^>50%), pooled estimates and confidence intervals were calculated with a random-effects model (DerSimonian Laird method). Otherwise, a fixed-effect model was used for meta-analysis. The primary analysis concerns any regular aspirin use (≥ 2 times per week). Wherever data were available, a set of sub-group analyses were performed to examine the effect of high frequency (daily) and long duration (≥ 5 years) aspirin use. The sub-group analyses were conducted to explore the influence of exposure variations on cancer risk. For cancer-specific mortality, the analyses were stratified into pre-diagnostic and post-diagnostic subgroups according to the use of aspirin before and after cancer diagnosis, respectively. All statistical analyses were conducted in R (version 4.0.3) and all p values were two-tailed.

### Dose-Response Analysis

To examine the potential non-linear trend of aspirin use and cancer risk, a dose-response analysis was performed. Since different units were used to measure aspirin use across various studies, aspirin use was converted into mg/day as a unified measurement. For each study, the daily aspirin dose was assigned to the corresponding RR estimate. The mid-point of the upper and lower boundaries in each category was assigned if the exact measurements were not available. The analysis was restricted to cancers for which the maximum aspirin dose was more than 300 mg/day in order to avoid a narrow dose range, which could lead to unreliable results for dose-response analysis. Non-linear regression was used to fit data to a model that defined the response (RR of developing a certain cancer) as a function of dose (aspirin use measurement). R square was calculated to quantify the goodness of fit and *P*<0.05 was considered statistically significant. Dose-response curves were used to present the dose-response relationships between aspirin use and cancer risk.

### Credibility Assessment

Cancer outcomes having statistically significant associations with aspirin use (*P*<0.05) were further classified into four categories (Class I, II, III, IV) (**Supplementary Box 1**) based on previously proposed criteria to assess the evidence credibility (20, 21). Small study effects were assessed with Egger’s regression asymmetry test (significance threshold *P*<0.10) (22). Potential excess significance bias was detected by evaluating whether the observed number of studies with nominally statistically significant results (*P*<0.10) was greater than the expected number of studies with statistically significant results (23–25). The detailed descriptions of these metrics/tests and their rationale are presented in **Supplementary Methods**. For outcomes that were investigated in both the meta-analysis of cohort studies and the meta-analysis of RCTs, the direction and statistical significance of the estimates were compared across the meta-analyses.

## Results

### Literature Review

**Figure 1:**
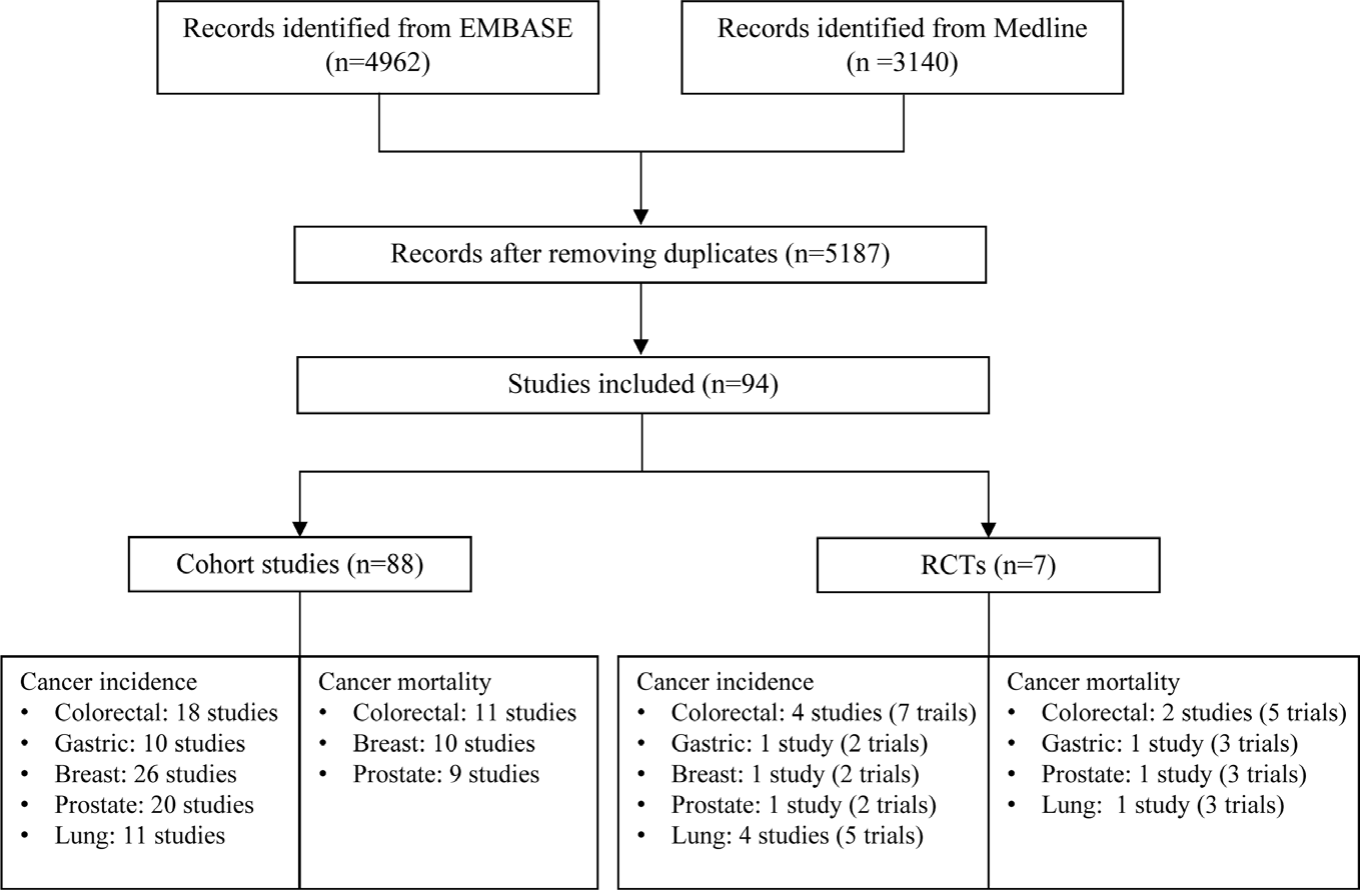
Flow chart of study selection for meta-analysis. For RCTs, number of studies represents the total number of studies included in the meta-analysis for the specific cancer and number of trials is the total number of clinical trials included in the meta-analysis. The inconsistency between these two numbers is due to that some studies used individual patient data from multiple trials for analysis.

A total of 5,187 articles were retrieved from two databases. After screening of title, abstract and full text, 94 eligible articles were finally included (**Figure 1**). Of them, 88 cohort studies summarized the associations of aspirin use with eight cancer outcomes (CRC incidence/mortality, gastric cancer incidence, breast cancer incidence/mortality, prostate cancer incidence/mortality and lung cancer incidence), and seven RCTs examined the effect of aspirin use on nine cancer outcomes (CRC incidence/mortality, gastric cancer incidence/mortality, breast cancer incidence, prostate cancer incidence/mortality and lung cancer incidence/mortality). There was one study with a combination of RCT and cohort design resulting the total number of 94 unique studies included for meta-analysis. The overall effects of aspirin use on cancer outcomes are summarized in **Tables 1**, **2** and main characteristics of included studies are presented in **Supplementary Tables 2, 3**.

**Table 1:** Up-to-date meta-analyses of cohort studies for 8 cancer outcomes and classification of evidence credibility.

Outcomes	Aspirin Use	Number of Studies	Events	Sample Size	Estimates (95% CI)	P	I^2^ (%)	95% PI	P_Egger_	P _Excess_	Class
**Cancer Incidence**											
CRC	Regular	18	127,291	3,536,448	0.85 (0.78-0.92)	9.26E-05	92	0.62-1.16	0.074	0.014	III
	Daily	9	57,727	753,690	0.85 (0.76-0.96)	0.010	86	0.62-1.17	0.035	0.211	IV
	≥5 yrs	6	17,101	1,994,676	0.76 (0.60-0.98)	0.032	84	0.42-1.37	0.105	NP	IV
Gastric cancer	Regular	10	14,933	2,378,794	0.67 (0.52-0.87)	0.003	96	0.30-1.52	0.954	NP	IV
	Daily	2	4,788	488,835	0.79 (0.53-1.18)	0.251	88	0.41-4.54	-	0.004	NS
	≥5 yrs	3	6,164	890,956	0.60 (0.38-0.94)	0.027	86	0.26-1.39	0.0002	NP	IV
Breast cancer	Regular	26	31,442	2,037,666	0.93 (0.87-0.99)	0.021	79	0.71-1.21	0.139	NP	IV
	Daily	11	7,247	386,589	0.94 (0.86-1.03)	0.183	70	0.75-1.18	0.045	0.517	NS
	≥5 yrs	13	18,443	1,244,134	1.03 (0.98-1.09)	0.188	36	0.85-1.18	0.012	NP	NS
Prostate cancer	Regular	20	81,485	2,093,539	0.93 (0.88-0.97)	0.002	83	0.78-1.09	0.103	NP	IV
	Daily	7	10,335	254,315	0.99 (0.93-1.05)	0.702	51	0.87-1.12	0.633	0.519	NS
	≥5 yrs	11	20,428	1,507,034	0.72 (0.47-1.10)	0.125	99	0.17-2.99	0.584	NP	NS
Lung cancer	Regular	11	37,451	1,907,323	0.95 (0.81-1.12)	0.568	96	0.57-1.60	0.635	NP	NS
	Daily	5	1,885	185,781	1.05 (1.01-1.09)	0.014	22	0.95-1.14	0.727	0.521	IV
	≥5 yrs	4	26,435	898,077	0.90 (0.67-1.21)	0.471	92	0.48-1.68	<0.0001	NP	NS
**Cancer-specific Mortality**											
CRC	Pre-diagnostic	6	14,430	711,160	0.78 (0.56-1.09)	0.148	98	0.33-1.84	0.629	NP	NS
	Post-diagnostic	8	11,152	148,214	0.83 (0.71-0.97)	0.023	75	0.55-1.25	0.005	NP	IV
Breast cancer	Pre-diagnostic	5	3,030	45,725	0.91 (0.83-1.01)	0.082	0	0.83-1.01	0.569	0.013	NS
	Post-diagnostic	8	5,237	62,684	0.81 (0.65-1.00)	0.049	79	0.46-1.40	0.172	NP	IV
Prostate cancer	Pre-diagnostic	3	1,657	34,245	0.94 (0.83-1.08)	0.395	0	0.83-1.08	0.552	NP	NS
	Post-diagnostic	7	4,521	103,811	0.87 (0.67-1.14)	0.312	89	0.44-1.71	0.018	NP	NS

CRC, colorectal cancer; yrs, years; PI, prediction interval; NS, not significant; NP, not pertinent (because the number of expected significant studies was larger than the number of observed significant studies).

**Table 2:** Up-to-date meta-analyses of RCTs for 9 cancer outcomes and classification of evidence credibility.

Outcomes	Number of Studies	Number of Trials	Events	Sample Size	Estimates (95% CI)	P	I^2^ (%)	95% PI	P_Egger_	P _Excess_	Class
**Cancer Incidence**											
CRC	4	7	902	81,119	0.74 (0.56-0.97)	0.031	73	0.43-1.27	0.248	0.154	IV
Gastric cancer	1	2 (IPD)	46	6,076	1.01 (0.54-1.86)	0.990	-	-	-	-	NS
Breast cancer	1	2 (IPD)	12	6,076	0.90 (0.26-3.07)	0.860	-	-	-	-	NS
Prostate cancer	1	2 (IPD)	313	6,076	0.87 (0.69-1.10)	0.250	-	-	-	-	NS
Lung cancer	4	5	756	73,222	0.98 (0.84-1.13)	0.757	0	0.84-1.13	0.179	0.220	NS
**Cancer-specific Mortality**											
CRC	2	5	292	19,172	0.63 (0.49-0.80)	2.01E-04	2	0.49-0.81	0.312	0.307	IV
Gastric cancer	1	3 (IPD)	71	10,502	0.69 (0.43-1.10)	0.110	-	-	-	-	NS
Prostate cancer	1	3 (IPD)	210	10,502	0.81 (0.61-1.06)	0.120	-	-	-	-	NS
Lung cancer	1	3 (IPD)	326	10,502	0.71 (0.58-0.89)	0.002	-	-	-	-	IV

Number of studies represents the total number of studies included in the meta-analysis for the specific cancer. Number of trials is the total number of clinical trials included in the meta-analysis. The inconsistency between these two numbers is due to that some studies used individual patient data from multiple trials for analysis. CRC, colorectal cancer; IPD, individual patient data; PI, prediction interval; NS, not significant.

### Up-to-Date Meta-Analyses of Cohort Studies and Evidence Assessment

**Figure 2:**
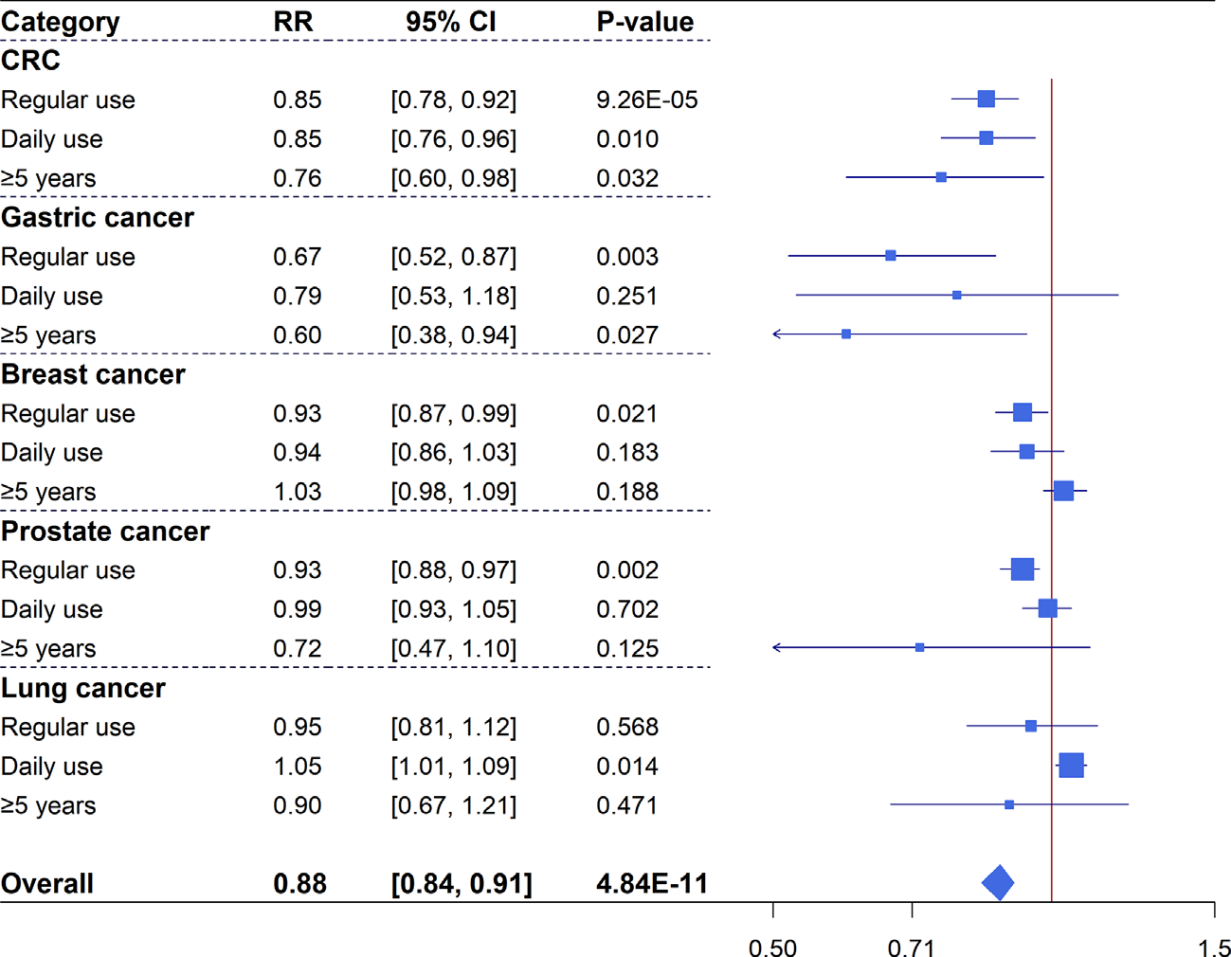
Forest plot of aspirin use in different categories and risk of common cancers in cohort studies.

***Colorectal cancer***: 18 studies with a total of 127,291 events and 3,536,448 participants were included for the meta-analysis of CRC incidence. Overall, there was an estimate of 15% reduction in CRC risk (18 studies, RR=0.85, 95%CI: 0.78-0.92, *P*=9.26×10^-5^) for any regular aspirin use, and significant associations were also identified for daily use (nine studies, RR=0.85, 95%CI: 0.76-0.96, *P*=0.010) and long-duration use (six studies, RR=0.76, 95%CI: 0.60-0.98, *P*=0.032). Considerable heterogeneity (I^2^>50%) was observed for these summary estimates and none of them had a 95% prediction interval (PI) excluding the null value. Excessive significance bias and small study effects were indicated for the summary estimate of any regular use (**Figure 2** and **Supplementary Figure 1**).

***Gastric cancer***: 10 studies were identified for the meta-analysis of gastric cancer with 14,933 events and 2,378,794 participants. An overall 33% reduced risk (10 studies, RR=0.67, 95%CI: 0.52-0.87, *P*=0.003) in gastric cancer was observed for regular aspirin use with high heterogeneity but no hints of biases (**Supplementary Figure 2**). Subgroup analysis of long-duration use showed a significant association (three studies, RR=0.60, 95%CI: 0.38-0.94, P=0.027). However, when analysis was restricted to daily aspirin use (two studies), a non-significant association was identified (RR=0.79, 95%CI: 0.53-1.18, P=0.251).

***Breast cancer***: Meta-analysis for breast cancer included 26 studies with 31,442 events and 2,037,666 participants. The primary analysis of regular aspirin use showed a significantly reduced risk (26 studies, RR=0.93, 95%CI: 0.87-0.99, P=0.021) despite substantial heterogeneity. However, subgroup analyses of daily or long-duration aspirin use revealed non-significant summary estimates (**Supplementary Figure 3**).

***Prostate cancer***: 20 studies, with a total of 81,485 events and 2,093,539 participants, examined the association between aspirin use and prostate cancer risk. Regular aspirin use was associated with a significant reduction of 7% prostate cancer risk (20 studies, RR=0.93, 95%CI: 0.88-0.97, *P*=0.002) with no hints of biases but substantial heterogeneity. Meta-analyses of daily use (seven studies) and long-duration use (11 studies) revealed non-significant associations (**Supplementary Figure 4**).

***Lung cancer***: Meta-analysis of 11 studies with 37,451 cases and 1,907,323 participants found inconsistent results for the association between aspirin use and lung cancer risk. The results showed a significantly increased risk for daily use (five studies, RR=1.05, 95%CI: 1.01-1.09, *P*=0.014) with no heterogeneity and no hints of excessive significance bias, but analyses of regular (11 studies, RR=0.95, 95%CI: 0.81-1.12, *P*=0.568) and long-duration (four studies, RR=0.90, 95%CI: 0.67-1.21, *P*=0.471) aspirin use showed non-significant estimates (**Supplementary Figure 5**).

**Figure 3:**
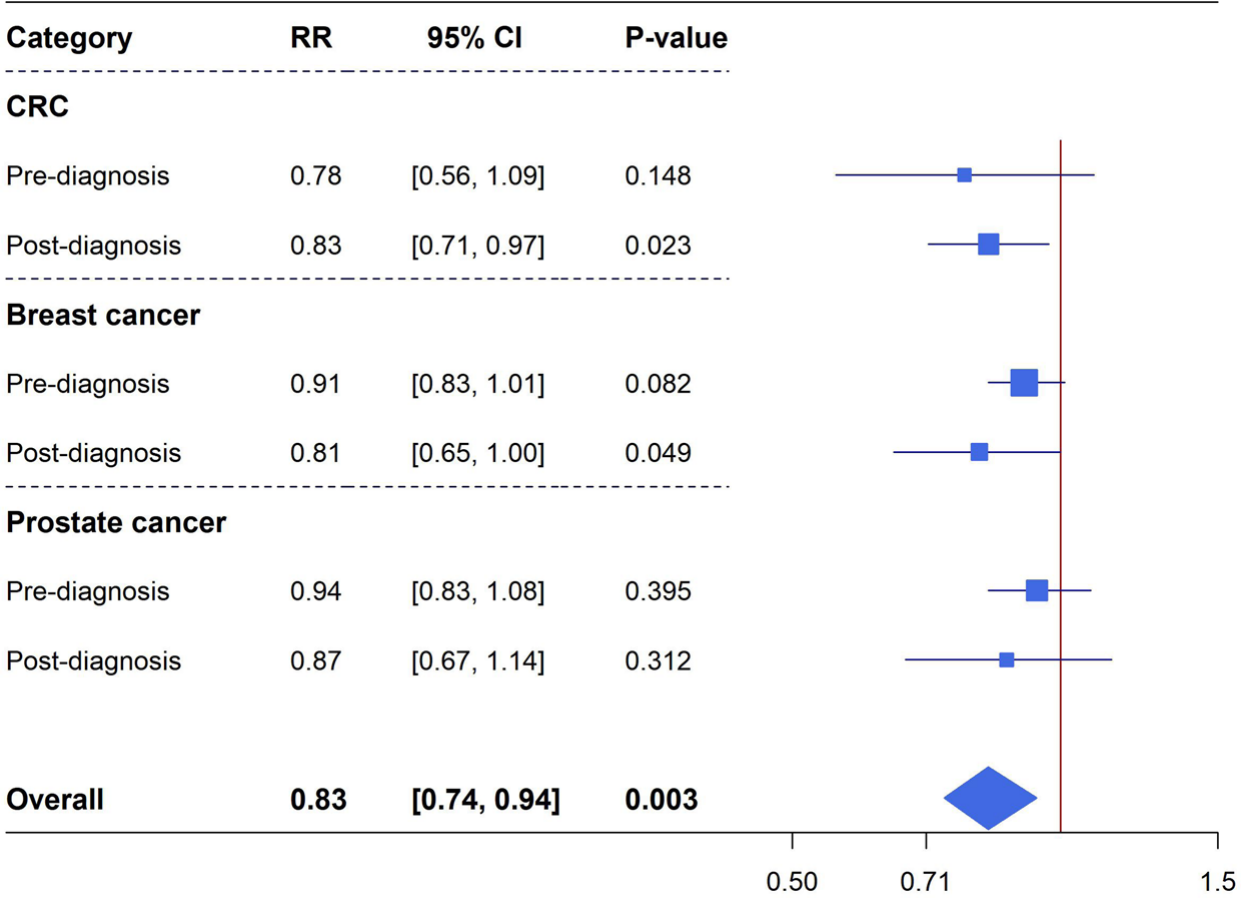
Forest plot of aspirin use in different categories and cancer specific mortality in cohort studies.

***Cancer mortality***: Meta-analyses of pre-diagnostic and post-diagnostic aspirin use on cancer-specific mortality in cancer patients were performed for CRC (totally 11 unique studies, six studies reporting data on pre-diagnosis use, eight studies reporting data on post-diagnosis use), breast cancer (10 unique studies in total, five studies reporting data on pre-diagnosis use, eight studies reporting data on post-diagnosis use) and prostate cancer (totally nine studies, three studies reporting data on pre-diagnosis use, seven studies reporting data on post-diagnosis use). There were no significant associations observed between pre-diagnosis aspirin use and CRC mortality (six studies, RR=0.78, 95%CI: 0.56-1.09, *P*=0.148), breast cancer mortality (five studies, RR=0.91, 95%CI: 0.83-1.01, *P*=0.082) and prostate cancer mortality (three studies, RR=0.94, 95%CI: 0.83-1.08, *P*=0.395). However, post-diagnostic aspirin use was identified to be significantly associated with a reduced risk of CRC mortality (eight studies, RR=0.83, 95%CI: 0.71-0.97, *P*=0.023) and breast cancer mortality (eight studies, RR=0.81, 95%CI: 0.65-1.00, *P*=0.049), these estimates have considerable heterogeneity but no excessive significant biases existed (**Figure 3** and **Supplementary Figures 6–8**).

The robustness of these summary estimates was then assessed using pre-defined evidence classification criteria (**Supplementary Box 1**). Due to the presence of considerable heterogeneity, excessive significance bias or small study effects, none of the observed associations qualified as convincing (Class I) or highly suggestive (Class II) evidence. Suggestive evidence (Class III) was presented for the reduced risk of CRC incidence (with any regular aspirin use). There was weak evidence (class IV) for the associations between aspirin use and the following outcomes: the reduced risk of CRC incidence (with daily and long-duration aspirin use) and mortality (aspirin use after cancer diagnosis), gastric cancer incidence (with any regular and long-duration aspirin use), breast cancer incidence (with any regular aspirin use) and mortality (aspirin use after cancer diagnosis), prostate cancer incidence (with any regular aspirin use), and the increased risk of lung cancer incidence (with daily aspirin use). The remaining assessed cancer outcomes presented non-significant summary estimates in relation to aspirin use.

### Meta-Analyses of RCTs and Evidence Assessment

**Figure 4:**
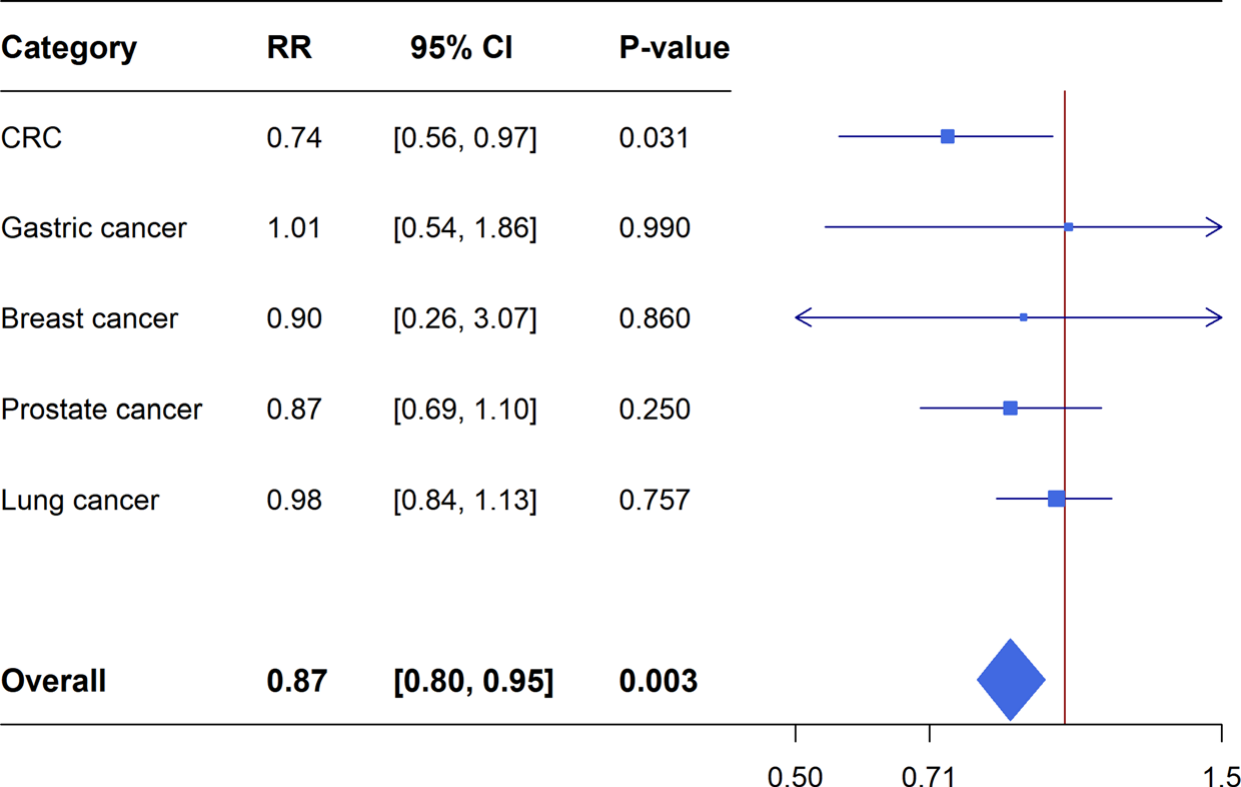
Forest plot of aspirin use in different categories and risk of common cancers in RCTs.

**Figure 5:**
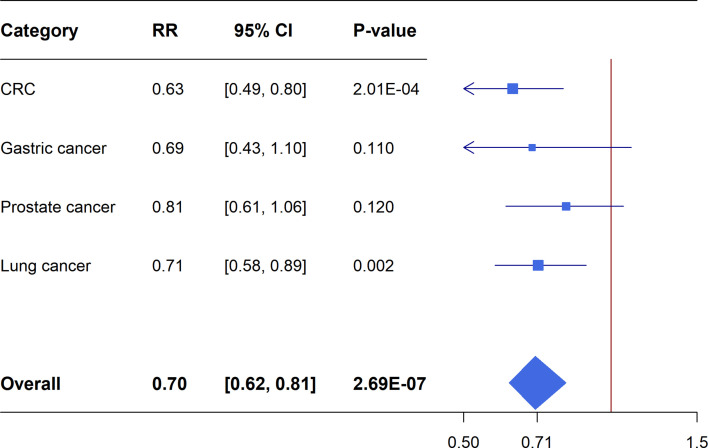
Forest plot of aspirin use in different categories and cancer specific mortality in RCTs.

Meta-analyses of the included RCTs showed statistically significant summary estimates (*P*<0.05) for CRC incidence (OR=0.74, 95%CI: 0.56-0.97; *P*=0.031), CRC mortality (OR=0.63, 95%CI: 0.49-0.80; *P*=2.01×10^-4^) and lung cancer mortality (OR=0.71, 95%CI: 0.58-0.89; *P*=0.002). Only one outcome (CRC mortality) had a statistically significant summary estimate with *P*<0.001, when correcting for the probability of a false positive (FDR) due to multiple comparisons. There was no effect on the risk of gastric cancer incidence/mortality, breast cancer incidence/mortality, prostate cancer incidence mortality and lung cancer incidence (**Figures 4**, **5**).

### Comparing Findings From Meta-Analyses of Cohort Studies and Meta-Analyses of RCTs

Overall, there were five common cancers with meta-analyses results available from both cohort studies and RCTs (**Table 3**). The meta-analyses of cohort studies and RCTs were consistent in showing that aspirin use had a protective effect on CRC risk (cohort studies: RR=0.85, 95%CI: 0.78-0.92; RCTs: RR=0.74, 95%CI: 0.56-0.97). Disagreement in either the direction or statistical significance of the summary estimates between meta-analyses of cohort studies and RCTs was seen for gastric, breast and prostate cancer. Both meta-analyses of cohort studies and RCTs showed non-significant summary estimates for lung cancer risk.

**Table 3:** Comparison of overlapping cancer outcomes examined in meta-analyses of cohort studies and RCTs.

Outcome	Meta-analyses of cohort studies		Meta-analyses of RCTs		Concordance*
	Events/Total	Estimates (95%CI)	Events/Total	Estimates (95%CI)	
Colorectal	127,291/3,536,448	0.85 (0.78-0.92)	902/81,119	0.74 (0.56-0.97)	Both S
Gastric	14,933/2,378,794	0.67 (0.52-0.87)	46/6,076	1.01 (0.54-1.86)	S Coh Only
Breast	31,442/2,037,666	0.93 (0.87-0.99)	12/6,076	0.90 (0.26-3.07)	S Coh Only
Prostate	81,485/2,093,539	0.93 (0.88-0.97)	313/6,076	0.87 (0.69-1.10)	S Coh Only
Lung	37,451/1,907,323	0.95 (0.81-1.12)	756/73,222	0.98 (0.84-1.13)	Both NS

*Both S, effects from meta-analyses of both cohort studies and randomized controlled trials are significant (P < 0.05) and of the same direction; Both NS, effects from meta-analyses of both cohort studies and randomized controlled trials are not significant (P > 0.05); S Coh Only, only effects from meta-analyses of cohort studies are significant (P < 0.05); Coh, cohort studies; RCT, randomized controlled trial.

### Dose-Response Analysis

**Figure 6:**
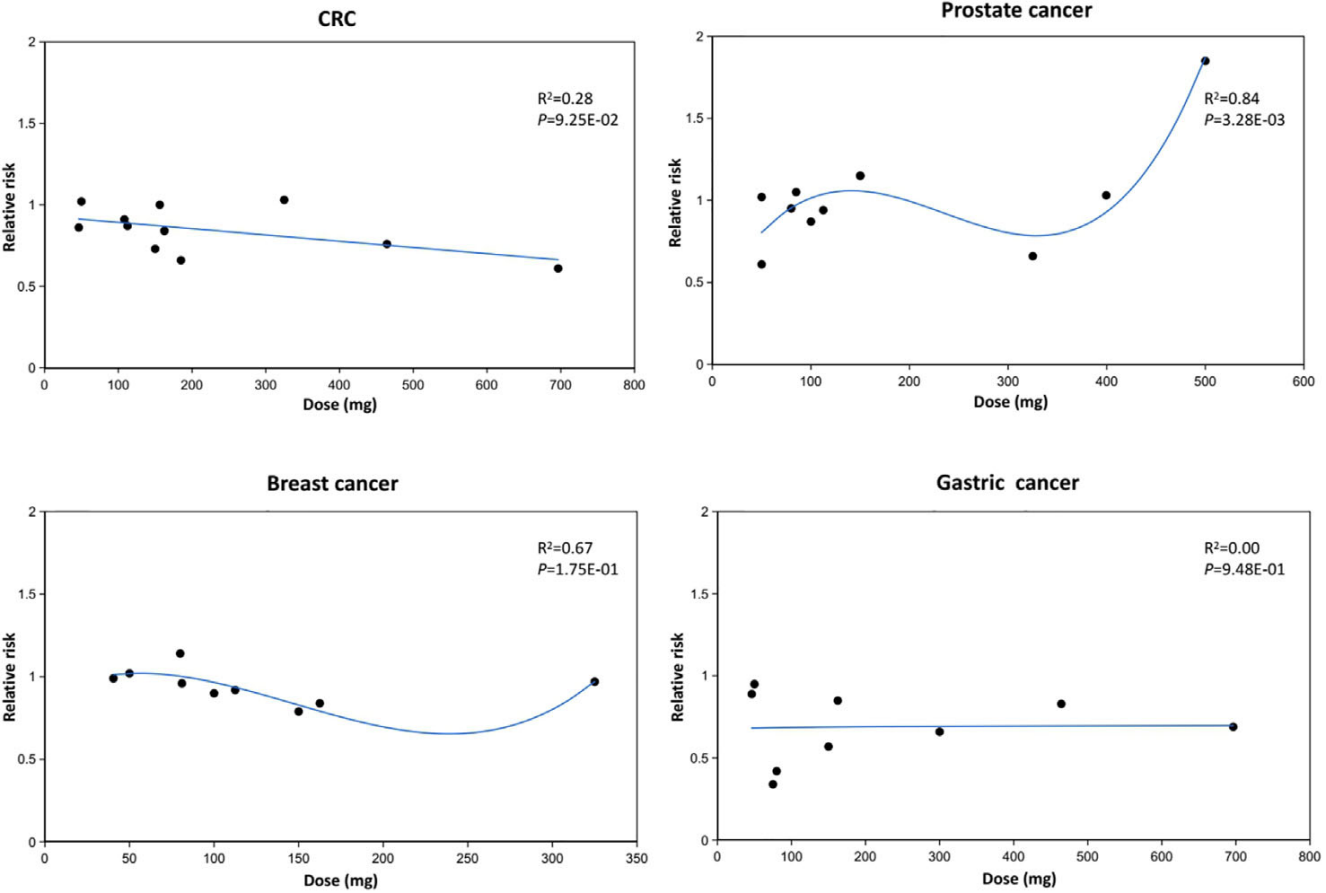
Dose response relationship between aspirin use and cancer risk.

**Figure 6** presents the dose-response relationship of aspirin use with cancer risk. The dose-response analysis indicated that the increment in the dose of aspirin was inversely associated with CRC risk, and this is consistent with the result that regular aspirin use was associated with a 15% lower risk in CRC (RR=0.85, 95%CI: 0.78-0.92) (**Supplementary Figure 1**). There was a non-linear relationship between aspirin dose and prostate cancer risk. The estimated RRs of developing prostate cancer reduced with the increment of aspirin dose and reached 0.66 (0.50-0.85) at the amount of 325 mg/day, but the inverse relationship was attenuated gradually for an aspirin dose higher than 325 mg/day and reached 1.85 (1.04-3.32) at the amount of 500 mg/day. The regression model indicated high goodness of fit for prostate cancer risk (R^2^ = 0.84, *P*=0.003). The potential non-linear trends of aspirin use and risk of breast cancer were similar to that of prostate cancer despite the fact that the regression model might not be highly fitted (R^2^ = 0.67, *P*=0.175). Lastly, there was no significant association between aspirin dose and gastric cancer risk.

## Discussion

In this study, a comprehensive overview of the associations between aspirin use and multiple cancer outcomes was presented. In total, 88 cohort studies that explored the associations between aspirin use and five common cancers were identified. Nominally significant associations with aspirin use were reported for a number of cancer outcomes: CRC incidence/mortality, gastric cancer incidence, breast cancer incidence/mortality, prostate cancer incidence and lung cancer incidence. An additional seven RCTs that investigated the effects of aspirin intake on these five cancers were identified. In these studies, significant effects were observed on CRC incidence/mortality and lung cancer mortality. When these two types of evidence were compared, both of them showed a protective effect of aspirin use on CRC risk, inconsistent evidence for the risk of gastric, breast and prostate cancer, and a non-significant effect on the risk of lung cancer.

The protective effect of aspirin use against CRC was validated in this meta-analysis. Evidence from the meta-analysis of cohort studies reported a 15% reduction of CRC incidence in regular and daily aspirin users, and a 24% reduction in long-duration users. Evidence from the meta-analysis of RCTs showed that aspirin reduced the risk of CRC incidence and mortality after a latency period of at least 10 years with a scheduled treatment of more than five years. The consistency of the direction and statistical significance of the estimates from cohort studies and RCTs supports the protective effect of aspirin on CRC risk. In addition, dose-response analysis showed a monotonically decreasing relationship between aspirin use and CRC risk, where the risk of CRC decreased with the increasing dose of aspirin intake. For cancers in other sites of the digestive tract, a significant association between aspirin use and reduced risk of gastric cancer was reported in meta-analyses of cohort studies with a lower level of evidence class (Class IV). However, the meta-analysis of RCTs showed a non-significant effect estimate for gastric cancer risk even after a long period of follow-up. Evidence from previous studies supporting the association of aspirin use with reduced gastric cancer risk was also less consistent. A significant inverse association between aspirin use and gastric cancer risk was reported in meta-analyses of observational studies (26–28). while the pooled meta-analysis conducted by Yang et al. reported a non-significant effect estimate (29). Given that aspirin may cause gastrointestinal bleeding, it is possible that patients with early symptoms of gastric cancer avoid using this drug, thus leading to an inverse association as reported.

Evidence from the meta-analysis of cohort studies links aspirin use to a lower risk of prostate cancer, and this is consistent with findings from previous studies. Huang et al. conducted a meta-analysis of 24 epidemiology studies and identified that aspirin use was associated with a reduced risk of prostate cancer, both in overall and cohort studies (30). However, half of the included studies were case-control studies, which may lead to potential recall and selection bias. The present study only included cohort studies and therefore likely has avoided the bias frequently introduced by case-control studies. Dose-response analysis revealed that extra-strength aspirin use appeared positively associated with prostate cancer incidence, suggesting that the protective effect of aspirin may be confined to low-dose use. Thus, further studies are warranted to validate this finding and to find the minimum effective dose required for prostate cancer prevention. Beyond the examination of cancer incidence, Zhou et al. performed a meta-analysis to explore the relationship between aspirin use and prostate cancer specific mortality; in that analysis, no significant association was identified (31). Liu et al. reported contrasting results and found that aspirin use was related to a modest reduction in prostate cancer specific mortality (32). Considering that variations in the timing of use may influence the effect of aspirin on cancer prognosis, the analyses were stratified into pre-diagnostic use and post-diagnostic use. Results showed that neither aspirin use before or after diagnosis was associated with prostate cancer specific mortality.

Both meta-analyses of cohort studies and RCTs found no significant association between regular aspirin use and lung cancer risk, and this is consistent with findings from previous meta-analyses. Jiang’s study identified the protective effect of aspirin regular use on lung cancer, but the association was mostly influenced by case-control studies instead of cohort studies (33). Similarly, Oh et al. found a significant association between aspirin use and the reduced risk of lung cancer by pooling data from case-control studies, but no association was identified among cohort studies (34). When analysis was restricted to daily use, results showed that aspirin use was significantly associated with an increased risk of lung cancer. Although previous findings suggested that regular aspirin use might result in higher reduction of lung cancer incidence, information was still needed about a number of modifiable factors, including aspirin dose, frequency, duration of use and timing of use. Whether these factors have an impact on the observed association and how they exert their effects still needs further investigation.

Taken all together, this up-to-date meta-analysis of cohort studies identified a number of nominally significant associations between aspirin use and cancer outcomes, but the evidence strength was also limited by the existence of considerable heterogeneity, the presence of excessive significance bias, and small study effects. Traditionally, RCTs are the gold standard for judging the benefits of treatment. Nevertheless, it should be acknowledged that statistical significance testing in a single meta-analysis gives only a partial picture, and multiple testing correction should be considered. To reduce the possibility of false positives, a strict *P* value threshold (*P*<10^-3^) was used to assess the evidence strength from meta-analysis RCTs. Using this approach, only CRC passed the threshold in the credibility assessment.

There are several biological mechanisms through which aspirin may exert its anti-cancer effect. It has been well characterized that aspirin could inhibit the activity of the enzyme cyclooxygenase 2 (COX-2) (35). COX-2 is a critical component of the inflammatory response in human body and prolonged inflammation can promote changes in cells that cause them to become malignant. This appears to be particularly true in CRC where inflammation can promote changes in cells that line the lower gastrointestinal tract, leading to the formation of precancerous growths (35, 36). Another potential mechanism for aspirin’s chemoprevention effect is the inhibition of NF-κB (36). NF-κB is a known COX-independent target that could directly interact with aspirin. The NF-κB signaling pathway plays an important role in promoting inflammatory responses and angiogenesis, thus inhibition of this pathway may contribute to the observed anti-cancer effects. However, Stark et al. identified that aspirin led to apoptosis in human CRC models by activating the NF-κB signaling pathway (37). It is possible that this differential effect may be related to the specific cell types and tissue environments. Other mechanisms that are independent of COX such as induction of gene selection (38), modulation of mitochondrial voltage dependent anion channels (VDACs) (39), and induction of polyamine catabolism (40), have also been proposed.

This study has several limitations. First, although cohort studies are less prone to recall or selection bias than case-control studies, they generally collect data only at baseline and lack information on exposure changes over time, thus causing possible misclassification of aspirin exposure. Second, there was considerable heterogeneity across the included studies due to differences in the investigated populations, baseline cancer risks, the definitions of exposure, assessment methods and adjusted confounders. Despite the fact that subgroup analysis could remove the heterogeneity to some extent, the strength of evidence was also limited due to the reduced number of included studies in each subgroup. Third, analyses only used summary estimates from studies fully published in Medline and Embase databases. It is well known that studies with negative results are less likely to be published than studies with positive results, and this publication bias may have led to an overestimation of the effect of aspirin use on cancer outcomes in this study.

## Conclusions

Overall, the present meta-analysis of cohort studies and RCTs provided evidence of a favorable effect of aspirin use on CRC risk, suggesting that aspirin may be considered an alternative drug for CRC prevention. This study provided limited evidence of an anti-cancer effect of aspirin use on other cancer types, including gastric, breast and prostate cancer. It was also demonstrated that there was a dose-response relationship of aspirin use with cancer risk, in which a high dose (>500mg/day) of aspirin use was significantly associated with an increased risk of prostate cancer. These findings should be further validated in large-scale cohort studies and prospective clinical trials.

## Data Availability Statement

The original contributions presented in the study are included in the article/**Supplementary Material**. Further inquiries can be directed to the corresponding authors.

## Author Contributions

LW and RZ: literature review. LW, LY, and RZ: data extraction. LW, JX, XZ, and XiL: statistical analysis. LW: manuscript preparation. XuL and PS: study conception, design, manuscript review and edit. All authors contributed to the article and approved the submitted version.

## Conflict of Interest

The authors declare that the research was conducted in the absence of any commercial or financial relationships that could be construed as a potential conflict of interest.
