# Supplementary material for: Aspirin Use and Common Cancer Risk: A Meta-Analysis of Cohort Studies and Randomized Controlled Trials
Source: Front Oncol. 2021 Jun 18;11:690219. doi: 10.3389/fonc.2021.690219 (PMC8279749; doi:10.3389/fonc.2021.690219)
Supplement: Supplementary file 1 [file DataSheet_1.docx]

**Supplementary method: metrics for evidence assessment**

(i) Summary estimates

We used the DL estimator for random-effects, since this is the usual default choice that has been used in previous meta-analyses and we wanted our estimates to be consistent with the existing literature. However, we should note that DL may tend to underestimate the 95% CI, especially when few studies are available and other methods and adjustments have been proposed in these cases (1).

(ii) Statistical significance and sample size

We used the following thresholds of statistical significance and sample size in the evidence classification: (a) Convincing (class I)/ Highly suggestive (class II): *P*<10^-6^ and more than 1,000 cases having the event of interest (or more than 20,000 participants for continuous outcomes); (b) Suggestive (class III): p-value <10^-3^ and more than 1,000 cases (or more than 20,000 participants for continuous outcomes); (c) Weak (class IV): *P*<0.05 and no restriction in relation to number of cases. These cut-offs are operational and the criteria should not be seen as absolute rules. A p-value of <10^-6^ corresponds to a Bayes factor (BF) of >25000 and a p-value of <10^-3^ corresponds to a BF of >50 (2). The BF provides a numerical value that quantifies how well the null hypothesis (H0) predicts the empirical data when compared to a competing hypothesis (H1). Therefore, a BF >50 indicates that the empirical data is 50 times more probable if H₁ were true than if H₀ were true and corresponds to a p-value of 0.001. Categorization of BF into levels of evidence indicates that a BF>50 suggests strong evidence against the null hypothesis, whereas a BF>25000 suggests very strong evidence (3, 4).

For sample size, the cut-offs are arbitrary. There has been evidence indicating that even with these very stringent p-values, false positive findings cannot be excluded (5). In addition, effect sizes tend to be inflated and false-positives are more common in smaller studies and with more limited evidence (6). Therefore, this set of cut-offs for the statistical significance and sample size were adopted to evaluate the credibility of these associations.

(iii) Heterogeneity among studies

The I^2^ metric (ranging from 0-100%) measures the ratio of true heterogeneity to total observed variation, and has been widely used to assess cross-study heterogeneity. The detailed method of calculating the I^2^ has been described elsewhere (7). Only meta-analyses with I^2^<50% were eligible to be classified as convincing (given that they also satisfied all the other criteria for class I evidence classification).

(iv) 95% prediction interval (PI)

This metric predicts the range of effect size that would be expected in a new original study, after accounting for both the heterogeneity among individual studies and the uncertainty of the summary effect as estimated in the random-effects model. The detailed method for calculating a prediction interval has been described in detail in previous publications (8). Only meta-analyses with a 95% prediction interval that excluded the null were eligible to be classified as convincing (given that they also satisfied all the other criteria for class I evidence classification).

(v) Small study effects

By using the Egger’s regression asymmetry test, we investigated if small studies tend to give larger estimates of effect size than large studies (significance threshold *P*<0.10) (9). Harbord et al. proposed a modified version of the Egger test for small-study effects in meta-analysis of controlled trials with binary endpoints (10). This test performed well in simulations of trials with little or no heterogeneity. The authors, in their summary, suggested that this modified Egger test had type I or II error rates lower than the Macaskill test but close to the original Egger test (11). Furthermore, the authors did not recommend the use of this test in meta-analyses of observational studies where there might be large imbalance in the group sizes and they also did not recommend if for trials with continuous outcomes. Therefore, we decided to use the original Egger test for analysis and only meta-analyses with no evidence of small study effects (*P*>0.10) were eligible to be classified as convincing (given that they also satisfied all the other criteria for class I evidence classification).

(vi) Excess significance

This test assesses if the observed number of studies with significant results is greater than the expected number (12). The observed number of studies with significant results was the number of component studies included in the meta-analysis with a statistically significant DL estimator (*P*<0.05) calculated in our study. The expected number of studies with significant findings was calculated by using the sum of statistical power estimated for each component study in the meta-analysis. To calculate the statistical power, we first assumed the effect size of the largest component study (with smallest variance) to be the best estimate of the true effect size, considering the fact that the random-effects estimate could be biased when selective reporting bias is present (13). The sample size and number of events were extracted from each component study. With a type I error of 0.05, we then estimated the *post hoc* statistical power for each component study of binary or time-to-event outcome using the approach described in previous publications (14). For continuous outcomes, the mean difference (MD) was first standardized and then transformed to odds ratio. To assess the difference between observed and expected number of studies with significant results, A χ2 test was conducted based on the statistic A = [(O−E)2/E+(O−E)2/(n−E)] (n: the total number of included studies; O: observed number of studies with significant results; E: expected number of studies with significant results). The detailed description of the excess significance test and statistical module (STATA) could be found at: http://www.dhe.med.uoi.gr/images/oldsite/software.html. This test has demonstrated limited power when few studies with observed significant results, and therefore, a lenient p-value threshold (*P*<0.10) is chosen (2). Only meta-analyses with no excess significance were eligible to be classified as convincing (given that they also satisfied all the other criteria for class I evidence classification).

**Reference**

1. Cornell JE, Mulrow CD, Localio R, Stack CB, Meibohm AR, Guallar E, et al. Random-effects meta-analysis of inconsistent effects: a time for change. *Annals of internal medicine* (2014) 160(4):267-70. doi: 10.7326/m13-2886

2. Zhou CK, Daugherty SE, Liao LM, Freedman ND, Abnet CC, Pfeiffer R, et al. Do Aspirin and Other NSAIDs Confer a Survival Benefit in Men Diagnosed with Prostate Cancer? A Pooled Analysis of NIH-AARP and PLCO Cohorts. *Cancer Prev Res (Phila)* (2017) 10(7):410-20. doi: 10.1158/1940-6207.CAPR-17-0033

3. Goodman SN. Toward evidence-based medical statistics. 2: The Bayes factor. *Annals of internal medicine* (1999) 130(12):1005-13. doi: 10.7326/0003-4819-130-12-199906150-00019

4. Hurwitz LM, Joshu CE, Barber JR, Prizment AE, Vitolins MZ, Jones MR, et al. Aspirin and Non-Aspirin NSAID Use and Prostate Cancer Incidence, Mortality, and Case Fatality in the Atherosclerosis Risk in Communities Study. *Cancer Epidemiol Biomarkers Prev* (2019) 28(3):563-9. doi: 10.1158/1055-9965.EPI-18-0965

5. Prasad V, Jena AB. Prespecified falsification end points: can they validate true observational associations? *Jama* (2013) 309(3):241-2. doi: 10.1001/jama.2012.96867

6. Ioannidis JP. Why most discovered true associations are inflated. *Epidemiology* (2008) 19(5):640-8. doi: 10.1097/EDE.0b013e31818131e7

7. Higgins JP, Thompson SG. Quantifying heterogeneity in a meta-analysis. *Stat Med* (2002) 21(11):1539-58. doi: 10.1002/sim.1186

8. Higgins JP, Thompson SG, Spiegelhalter DJ. A re-evaluation of random-effects meta-analysis. *J R Stat Soc Ser A Stat Soc* (2009) 172(1):137-59. doi: 10.1111/j.1467-985X.2008.00552.x

9. Egger M, Davey Smith G, Schneider M, Minder C. Bias in meta-analysis detected by a simple, graphical test. *BMJ* (1997) 315(7109):629-34. doi: 10.1136/bmj.315.7109.629

10. Harbord RM, Egger M, Sterne JA. A modified test for small-study effects in meta-analyses of controlled trials with binary endpoints. *Stat Med* (2006) 25(20):3443-57. doi: 10.1002/sim.2380

11. Macaskill P, Walter SD, Irwig L. A comparison of methods to detect publication bias in meta-analysis. *Stat Med* (2001) 20(4):641-54. doi: 10.1002/sim.698

12. Ioannidis JP. Excess significance bias in the literature on brain volume abnormalities. *Arch Gen Psychiatry* (2011) 68(8):773-80. doi: 10.1001/archgenpsychiatry.2011.28

13. Tsoi KK, Chan FC, Hirai HW, Sung JJ. Risk of gastrointestinal bleeding and benefit from colorectal cancer reduction from long-term use of low-dose aspirin: A retrospective study of 612 509 patients. *J Gastroenterol Hepatol* (2018) 33(10):1728-36. doi: 10.1111/jgh.14261

14. Ioannidis JP, Trikalinos TA. An exploratory test for an excess of significant findings. *Clin Trials* (2007) 4(3):245-53. doi: 10.1177/1740774507079441
