# Supplementary material for: Aspirin Use and Common Cancer Risk: A Meta-Analysis of Cohort Studies and Randomized Controlled Trials
Source: Front Oncol. 2021 Jun 18;11:690219. doi: 10.3389/fonc.2021.690219 (PMC8279749; doi:10.3389/fonc.2021.690219)
Supplement: Supplementary file 2 [file DataSheet_2.docx]

**Title: Aspirin use and common cancer risk: a meta-analysis of cohort studies and randomized controlled trials**

**Supplementary Figures**

**Supplementary Figure 1**. Forest plot of aspirin use and risk of CRC.

**Supplementary Figure 2**. Forest plot of aspirin use and risk of gastric cancer.

**Supplementary Figure 3**. Forest plot of aspirin use and risk of breast cancer.

**Supplementary Figure 4**. Forest plot of aspirin use and risk of prostate cancer.

**Supplementary Figure 5**. Forest plot of aspirin use and risk of lung cancer.

**Supplementary Figure 6**. Forest plot of aspirin use and CRC specific mortality.

**Supplementary Figure 7**. Forest plot of aspirin use and breast cancer specific mortality.

**Supplementary Figure 8**. Forest plot of aspirin use and prostate cancer specific mortality.


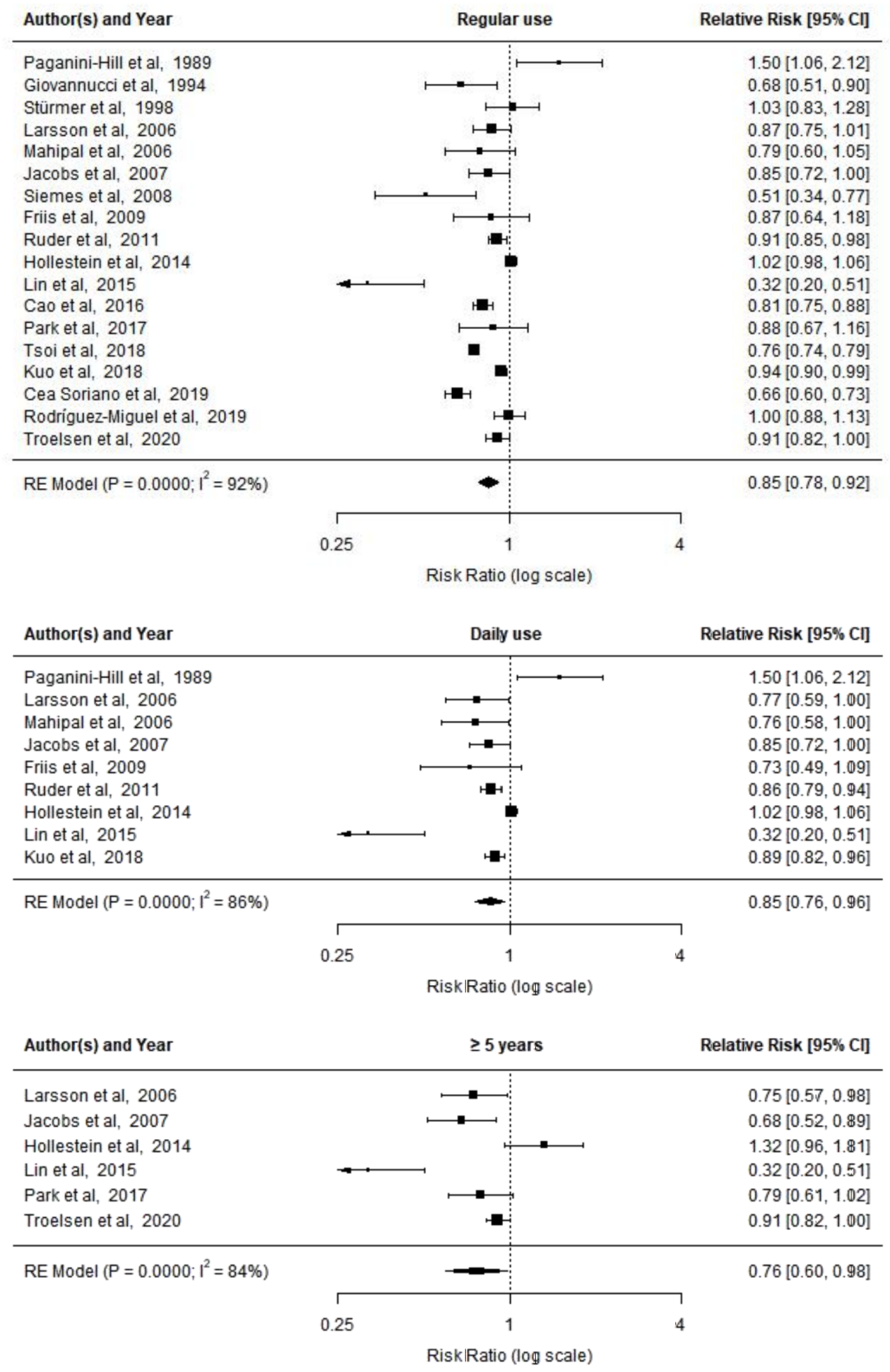


**Supplementary Figure 1**. Forest plot of aspirin use and risk of CRC.


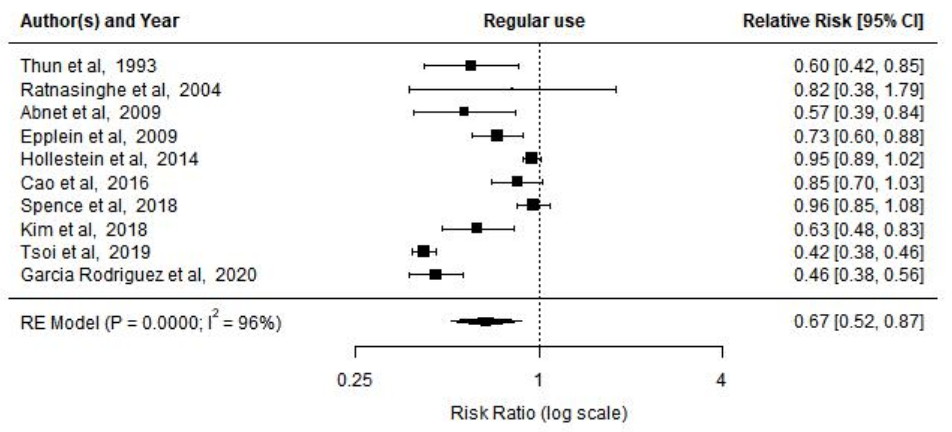


**Supplementary Figure 2**. Forest plot of aspirin use and risk of gastric cancer.


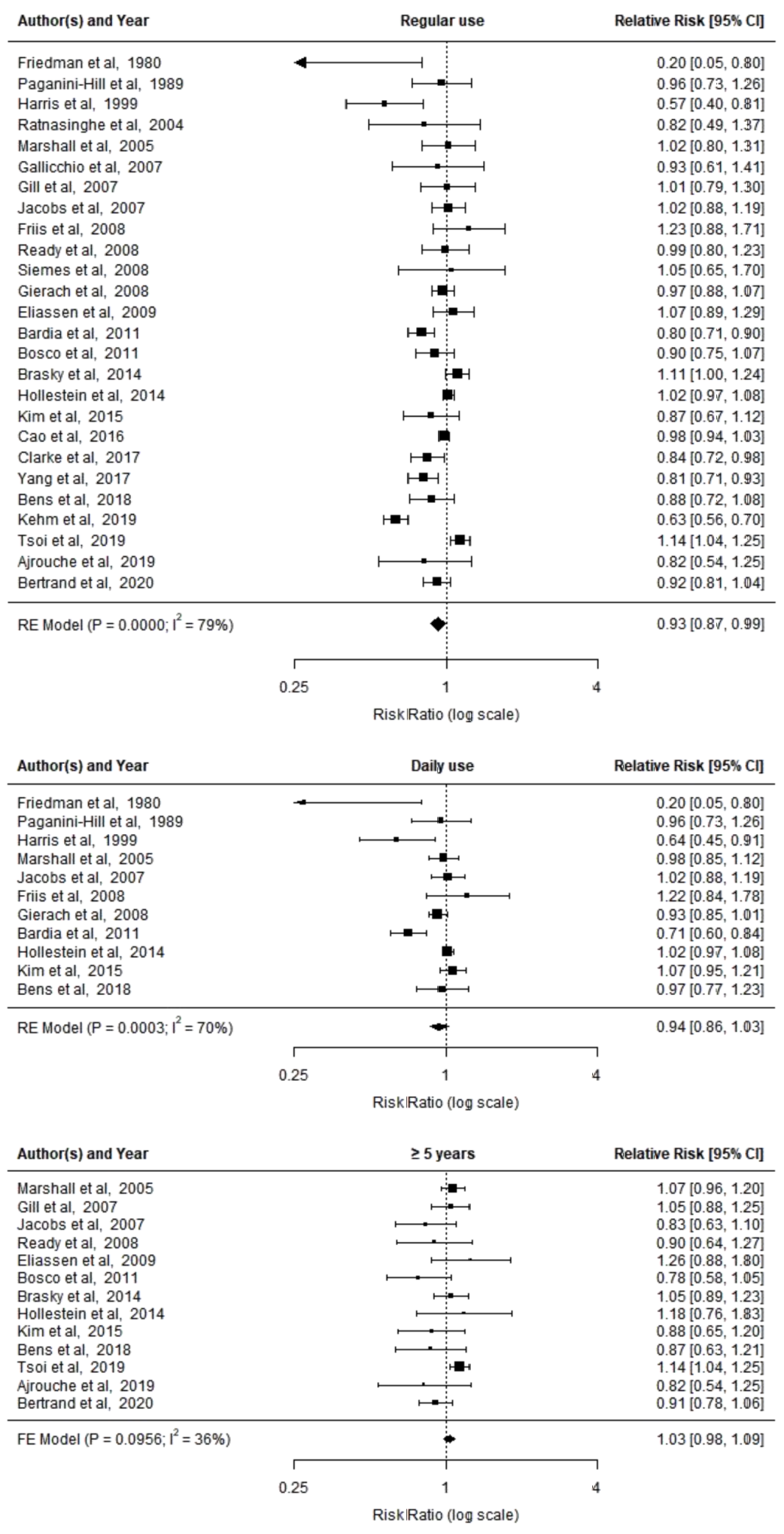


**Supplementary Figure 3**. Forest plot of aspirin use and risk of breast cancer.


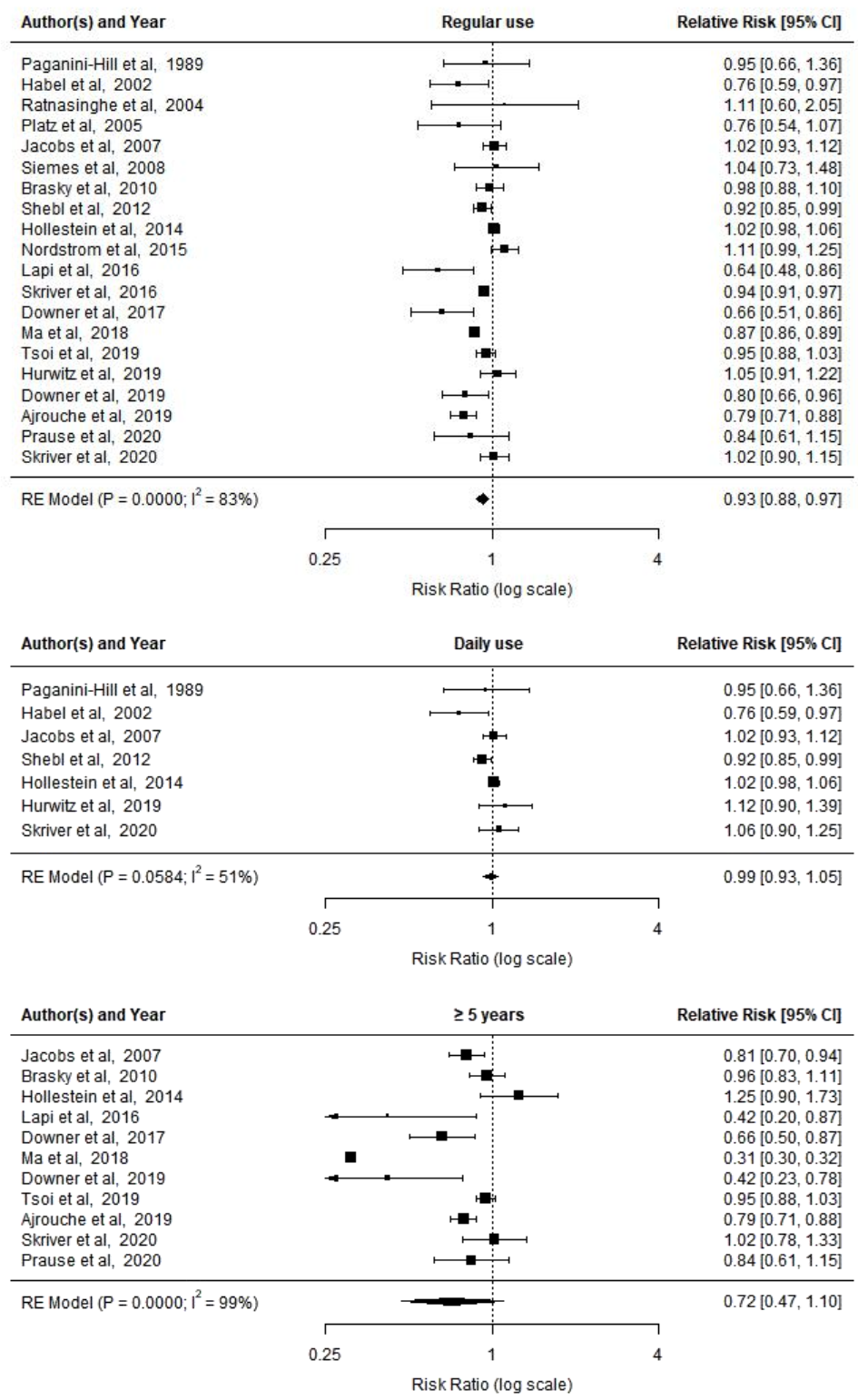


**Supplementary Figure 4**. Forest plot of aspirin use and risk of prostate cancer.


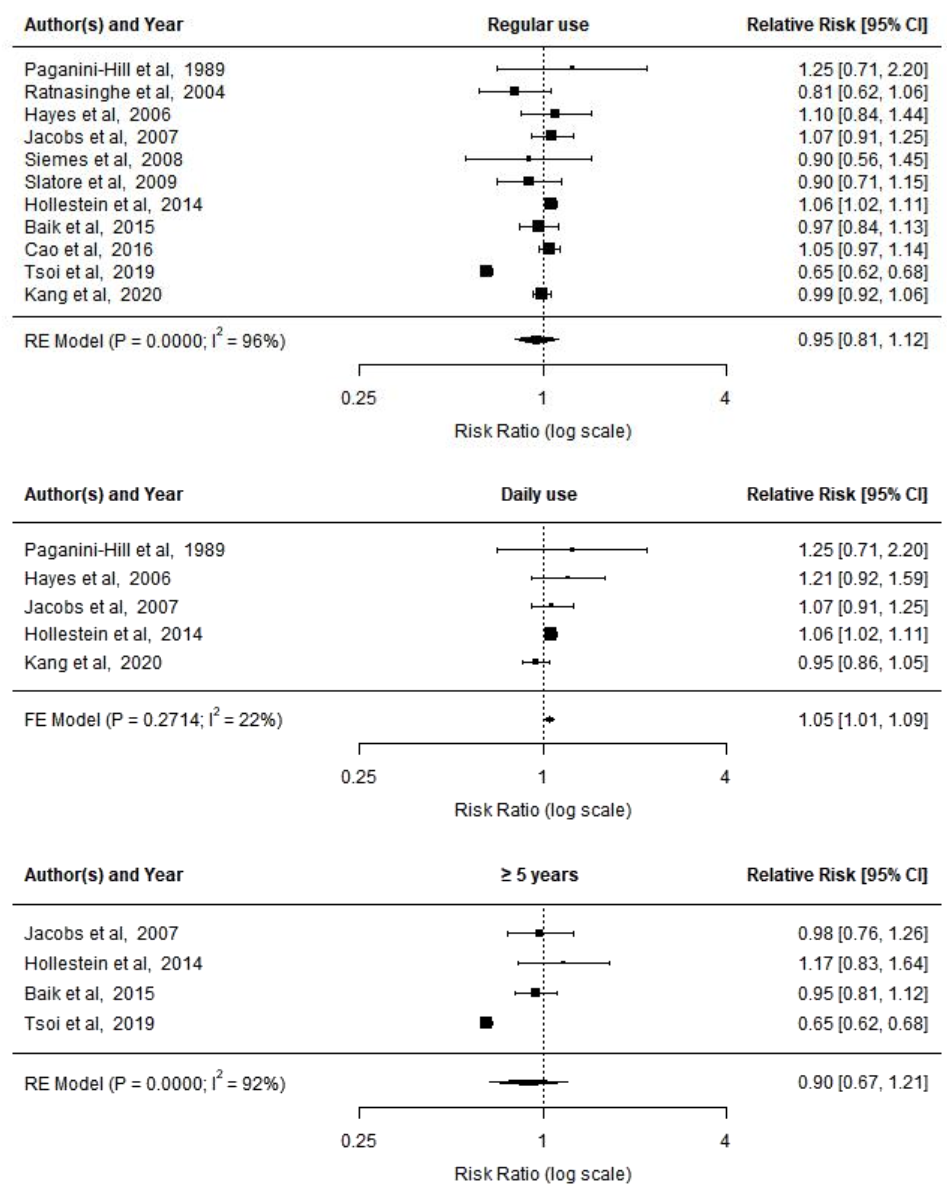


**Supplementary Figure 5**. Forest plot of aspirin use and risk of lung cancer.


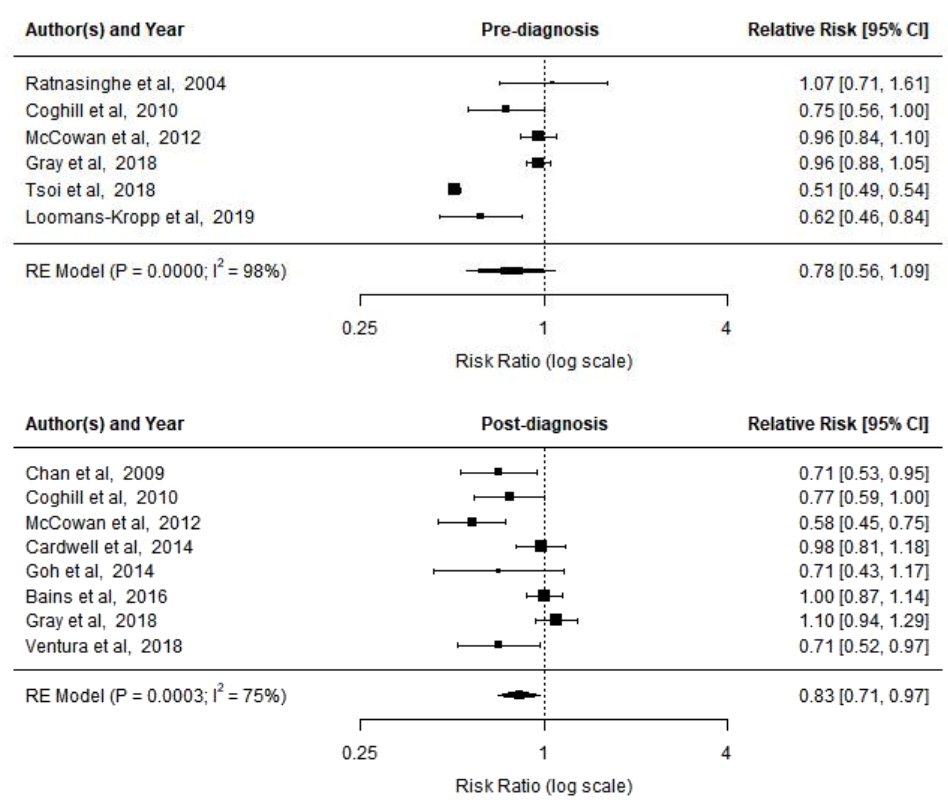


**Supplementary Figure 6**. Forest plot of aspirin use and CRC specific mortality.


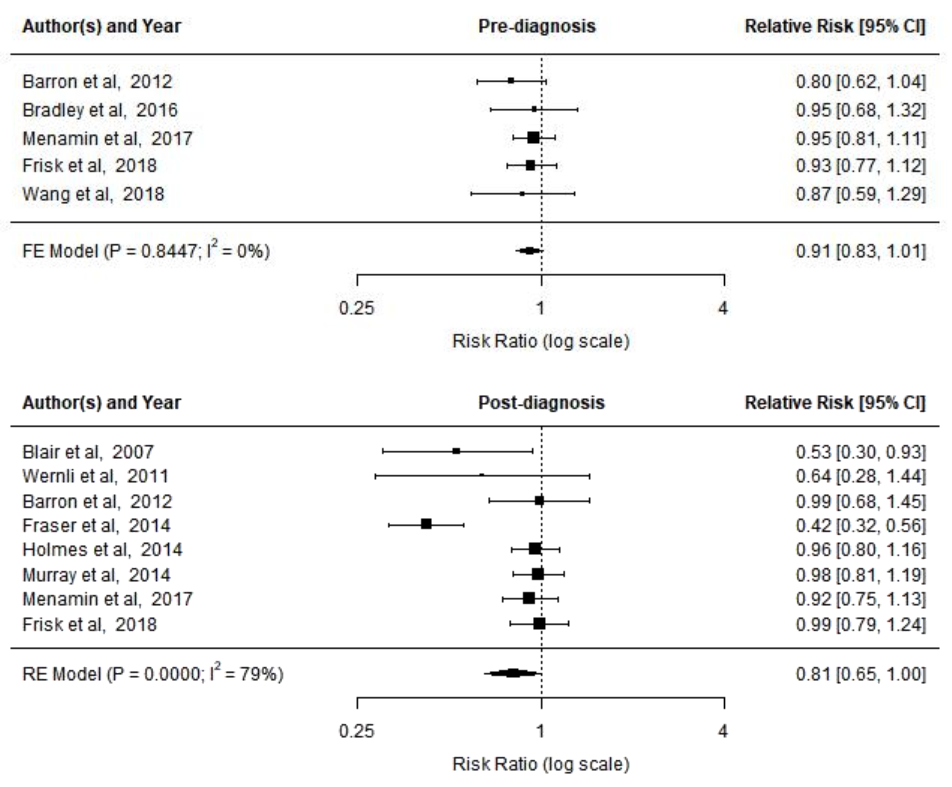


**Supplementary Figure 7**. Forest plot of aspirin use and breast cancer specific mortality.


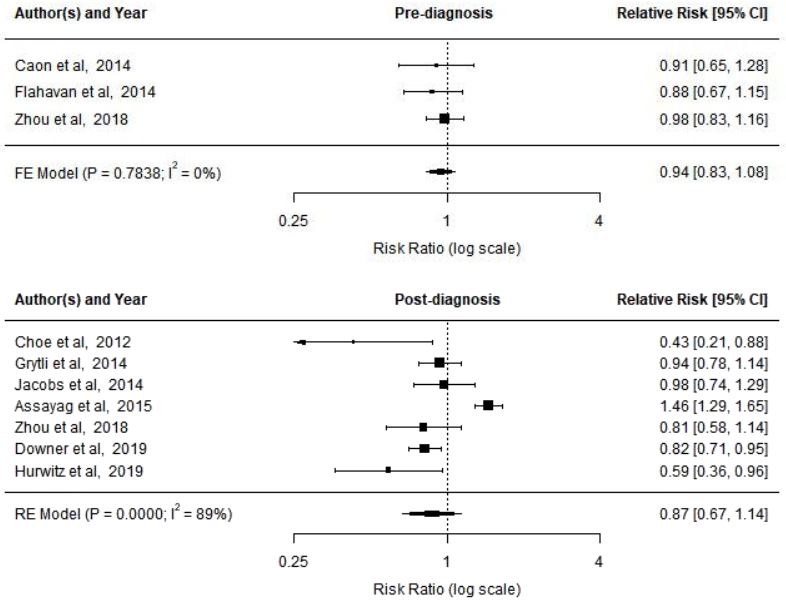


**Supplementary Figure 8**. Forest plot of aspirin use and prostate cancer specific mortality.
