# Supplementary material for: Aspirin Use and Common Cancer Risk: A Meta-Analysis of Cohort Studies and Randomized Controlled Trials
Source: Front Oncol. 2021 Jun 18;11:690219. doi: 10.3389/fonc.2021.690219 (PMC8279749; doi:10.3389/fonc.2021.690219)
Supplement: Supplementary file 3 [file DataSheet_3.docx]

**Title: Aspirin use and common cancer risk: a meta-analysis of cohort studies and randomized controlled trials**

**Supplementary Box 1. Credibility assessment criteria for meta-analyses of cohort studies and randomized controlled trials.**

| **Evidence class** | **Classification criteria** |
| --- | --- |
| Convincing (class I) | Associations with *P*<10^-6^; more than 1,000 cases having the event of interest (or more than 20,000 participants for continuous outcomes); the largest component study reporting a nominal statistically significant result (*P*<0.05); a 95% prediction interval that excluded the null; no large heterogeneity (I^2^<50%); no evidence of small study effect (*P*>0.10); no excess significance bias (*P*>0.10). |
| Highly suggestive(class II) | Associations with *P*<10^-6^, more than 1,000 cases (or more than 20,000 participants for continuous outcomes) and the largest component study reporting a statistically significant result (*P*<0.05). |
| Suggestive (class III) | Associations with *P*<10^-3^ and more than 1,000 cases (or more than 20,000 participants for continuous outcomes). |
| Weak (class IV) | Remaining statistically significant associations with *P*<0.05. |

**Supplementary Table 1. Search strategy.**

| **Search strategy** | **Databases: Ovid Medline(R), EMBASE**  **Last update:16 Oct 2020**  **Search limit: Humans** |
| --- | --- |
| 1. Aspirin terms | (Aspirin OR Aspirins OR “Acetyl Salicylic Acid” OR ASA OR “Acetylsalicylic Acid” OR Acetylsalicylic OR acetylsalicylate OR “salicylic acid” OR salicylate OR “2-(Acetyloxy)benzoic Acid” OR “2-Acetoxybenzoic Acid” OR “o-Acetylsalicylic Acid” OR “o-Acetoxybenzoic Acid” OR Acylpyrin OR Aloxiprimum OR Colfarit OR Dispril OR Easprin OR Ecotrin OR Endosprin OR Magnecyl OR Micristin OR Polopirin OR Polopiryna OR Solprin OR Solupsan OR Zorprin OR Acetysal).mp |
| 1. Cancer terms | (cancer OR cancers OR neoplasm OR neoplasms).mp |
| 1. Combination | 1 AND 2 |

**Supplementary Table 2. Basic characteristics and main findings for each study.**

| **Author, Year** | **Outcome** | **Population** | **Aspirin use** | **Events** | **Participants** | **Metric** | **Effect size (95% CI)** |
| --- | --- | --- | --- | --- | --- | --- | --- |
| Paganini-Hill et al, 1989(1) | CRC | General | Regular use | 153 | 11767 | RR | 1.50 (1.10-2.20) |
|  | CRC | General | Daily use | 153 | 11767 | RR | 1.50 (1.10-2.20) |
| Giovannucci et al, 1994(2) | CRC | Men | Regular use | 251 | 48084 | RR | 0.68 (0.52-0.92) |
| Stürmer et al, 1998(3) | CRC | Men | Regular use | 341 | 22071 | RR | 1.03 (0.83-1.28) |
| Larsson et al, 2006(4) | CRC | General | Regular use | 705 | 74250 | RR | 0.87 (0.75-1.01) |
|  | CRC | General | Daily use | 487 | 74024 | RR | 0.77 (0.59-0.99) |
|  | CRC | General | ≥ 5 yrs | 444 | 73981 | RR | 0.75 (0.58-0.99) |
| Mahipal et al, 2006(5) | CRC | Women | Regular use | 239 | 7844 | HR | 0.79 (0.59-1.04) |
|  | CRC | Women | Daily use | 255 | 6345 | HR | 0.76 (0.58-1.00) |
| Jacobs et al, 2007(6) | CRC | General | Regular use | 672 | 51407 | RR | 0.85 (0.72-1.00) |
|  | CRC | General | Daily use | 672 | 51407 | RR | 0.85 (0.72-1.00) |
|  | CRC | General | ≥ 5 yrs | 480 | 36193 | RR | 0.68 (0.52-0.90) |
| Siemes et al, 2008(7) | CRC | General | Regular use | 124 | 7621 | HR | 0.51 (0.34-0.77) |
| Friis et al, 2009(8) | CRC | General | Regular use | 504 | 47774 | RR | 0.87 (0.64-1.18) |
|  | CRC | General | Daily use | 482 | 47752 | RR | 0.73 (0.49-1.10) |
| Ruder et al, 2011(9) | CRC | General | Regular use | 3880 | 301240 | HR | 0.91 (0.85-0.98) |
|  | CRC | General | Daily use | 2072 | 301240 | HR | 0.86 (0.79-0.94) |
| Hollestein et al, 2014(10) | CRC | General | Regular use | 972 | 109276 | HR | 1.02 (0.98-1.06) |
|  | CRC | General | Daily use | 972 | 109276 | HR | 1.02 (0.98-1.06) |
|  | CRC | General | ≥ 5 yrs | 96 | 14612 | HR | 1.32 (0.96-1.80) |
| Lin et al, 2015(11) | CRC | General | Regular use | 277 | 60828 | HR | 0.32 (0.20-0.50) |
|  | CRC | General | Daily use | 335 | 47382 | HR | 0.32 (0.20-0.50) |
|  | CRC | General | ≥ 5 yrs | 25 | 37929 | HR | 0.32 (0.20-0.50) |
| Cao et al, 2016(12) | CRC | General | Regular use | 2895 | 135965 | RR | 0.81 (0.75-0.88) |
| Park et al, 2017(13) | CRC | General | Regular use | 3100 | 183199 | HR | 0.88 (0.67-1.16) |
|  | CRC | General | ≥ 5 yrs | 2547 | 183199 | HR | 0.79 (0.61-1.02) |
| Kuo et al, 2018(14) | CRC | General | Regular use | 62691 | 125327 | OR | 0.94 (0.90-0.99) |
|  | CRC | General | Daily use | 52299 | 104497 | OR | 0.89 (0.83-0.97) |
| Tsoi et al, 2018(15) | CRC | General | Regular use | 18454 | 612509 | OR | 0.76 (0.74-0.79) |
|  | CRC mortality | General | Pre-diagnostic | 9026 | 612509 | OR | 0.51 (0.49-0.54) |
| Cea Soriano et al, 2019(16) | CRC | General | Regular use | 3033 | 13033 | RR | 0.66 (0.60-0.73) |
| Rodríguez-Miguel et al, 2019(17) | CRC | General | Regular use | 15491 | 75491 | OR | 1.00 (0.88-1.13) |
| Troelsen et al, 2020(18) | CRC | General | Regular use | 13509 | 1648762 | RR | 0.91 (0.82-1.00) |
|  | CRC | General | ≥ 5 yrs | 13509 | 1648762 | RR | 0.91 (0.82-1.00) |
| Ratnasinghe et al, 2004(19) | CRC mortality | General | Pre-diagnostic | 193 | 22834 | RR | 1.07 (0.71-1.60) |
| Chan et al, 2009(20) | CRC mortality | General | Post-diagnostic | 222 | 1279 | RR | 0.71 (0.53-0.95) |
| Coghill et al, 2010(21) | CRC mortality | General | Pre-diagnostic | 316 | 1549 | RR | 0.75 (0.56-1.00) |
|  | CRC mortality | General | Post-diagnostic | 334 | 1549 | RR | 0.77 (0.59-1.00) |
| McCowan et al, 2012(22) | CRC mortality | General | Pre-diagnostic | 1047 | 3016 | RR | 0.96 (0.84-1.11) |
|  | CRC mortality | General | Post-diagnostic | 951 | 2920 | RR | 0.58 (0.45-0.75) |
| Cardwell et al, 2014(23) | CRC mortality | General | Post-diagnostic | 1559 | 9089 | RR | 0.98 (0.82-1.19) |
| Goh et al, 2014(24) | CRC mortality | General | Post-diagnostic | 181 | 726 | RR | 0.71 (0.43-1.16) |
| Bains et al, 2016(25) | CRC mortality | General | Post-diagnostic | 6533 | 23162 | RR | 1.00 (0.87-1.14) |
| Gray et al, 2018(26) | CRC mortality | General | Pre-diagnostic | 3655 | 10408 | RR | 0.96 (0.88-1.05) |
|  | CRC mortality | General | Post-diagnostic | 1064 | 8391 | RR | 1.10 (0.94-1.29) |
| Ventura et al, 2018(27) | CRC mortality | General | Post-diagnostic | 308 | 101098 | RR | 0.71 (0.52-0.97) |
| Loomans-Kropp et al, 2019(28) | CRC mortality | General | Pre-diagnostic | 193 | 60844 | RR | 0.62 (0.46-0.85) |
| Thun et al, 1993(29) | Gastric cancer | General | Regular use | 169 | 635031 | RR | 0.60 (0.42-0.85) |
| Ratnasinghe et al, 2004(19) | Gastric cancer | General | Regular use | 48 | 22834 | RR | 0.82 (0.38-1.81) |
| Abnet et al, 2009(30) | Gastric cancer | General | Regular use | 360 | 311115 | HR | 0.57 (0.39-0.85) |
| Epplein et al, 2009(31) | Gastric cancer | General | Regular use | 430 | 169292 | HR | 0.73 (0.61-0.89) |
|  | Gastric cancer | General | ≥ 5 yrs | 309 | 169171 | HR | 0.68 (0.50-0.93) |
| Hollestein et al, 2014(10) | Gastric cancer | General | Regular use | 268 | 109276 | HR | 0.95 (0.88-1.01) |
|  | Gastric cancer | General | Daily use | 268 | 109276 | HR | 0.95 (0.88-1.01) |
|  | Gastric cancer | General | ≥ 5 yrs | 28 | 109276 | HR | 0.88 (0.51-1.51) |
| Cao et al, 2016(12) | Gastric cancer | General | Regular use | 451 | 135965 | HR | 0.85 (0.70-1.03) |
| Spence et al, 2018(32) | Gastric cancer | General | Regular use | 2391 | 4654 | HR | 0.96 (0.85-1.08) |
| Kim et al, 2018(33) | Gastric cancer | General | Regular use | 4520 | 375039 | HR | 0.63 (0.48-0.83) |
|  | Gastric cancer | General | Daily use | 4520 | 379559 | HR | 0.63 (0.48-0.83) |
| Tsoi et al, 2019(34) | Gastric cancer | General | Regular use | 5827 | 612509 | RR | 0.42 (0.38-0.46) |
|  | Gastric cancer | General | ≥ 5 yrs | 5827 | 612509 | RR | 0.42 (0.38-0.46) |
| Garcia Rodriguez et al,  2020(35) | Gastric cancer | General | Regular use | 469 | 3079 | OR | 0.46 (0.38-0.57) |
| Friedman et al, 1980(36) | Breast cancer | Women | Regular use | NA | NA | RR | 0.20 (0.05-0.80) |
|  | Breast cancer | Women | Daily use | NA | NA | RR | 0.20 (0.05-0.80) |
| Paganini-Hill et al, 1989(1) | Breast cancer | Women | Regular use | 181 | 7220 | RR | 0.96 (0.73-1.25) |
|  | Breast cancer | Women | Daily use | 181 | 7401 | RR | 0.96 (0.73-1.25) |
| Harris et al, 1999(37) | Breast cancer | Women | Regular use | 276 | 19776 | RR | 0.57 (0.40-0.81) |
|  | Breast cancer | Women | Daily use | 280 | 19719 | RR | 0.64 (0.45-0.90) |
| Ratnasinghe et al, 2004(19) | Breast cancer | Women | Regular use | 131 | 12834 | RR | 0.82 (0.49-1.36) |
| Marshall et al, 2005(38) | Breast cancer | Women | Regular use | 2391 | 114640 | RR | 1.02 (0.80-1.31) |
|  | Breast cancer | Women | Daily use | NA | NA | RR | 0.98 (0.86-1.13) |
|  | Breast cancer | Women | ≥ 5 yrs | NA | NA | RR | 1.07 (0.96-1.20) |
| Gallicchio et al, 2007(39) | Breast cancer | Women | Regular use | 253 | 15651 | RR | 0.93 (0.61-1.41) |
| Gill et al, 2007(40) | Breast cancer | Women | Regular use | 914 | 54592 | HR | 1.01 (0.79-1.30) |
|  | Breast cancer | Women | ≥ 5 yrs | 1005 | 59466 | HR | 1.05 (0.88-1.25) |
| Jacobs et al, 2007(6) | Breast cancer | Women | Regular use | 835 | 50735 | RR | 1.02 (0.88-1.19) |
|  | Breast cancer | Women | Daily use | 835 | 25661 | RR | 1.02 (0.88-1.19) |
|  | Breast cancer | Women | ≥ 5 yrs | 626 | 18338 | RR | 0.83 (0.63-1.10) |
| Friis et al, 2008(41) | Breast cancer | Women | Regular use | 287 | 24439 | RR | 1.23 (0.89-1.72) |
|  | Breast cancer | Women | Daily use | 493 | 24439 | RR | 1.22 (0.80-1.70) |
| Gierach et al, 2008(42) | Breast cancer | Women | Regular use | 1752 | 124372 | RR | 0.97 (0.88-1.07) |
|  | Breast cancer | Women | Daily use | 774 | 126124 | RR | 0.93 (0.85-1.01) |
| Ready et al, 2008(43) | Breast cancer | Women | Regular use | 479 | 37676 | HR | 0.99 (0.80-1.23) |
|  | Breast cancer | Women | ≥ 5 yrs | 411 | 31756 | HR | 0.90 (0.64-1.27) |
| Siemes et al, 2008(7) | Breast cancer | Women | Regular use | 89 | 4560 | HR | 1.05 (0.65-1.71) |
| Eliassen et al, 2009(44) | Breast cancer | Women | Regular use | 1002 | 111290 | RR | 1.07 (0.89-1.29) |
|  | Breast cancer | Women | ≥ 5 yrs | 895 | 111290 | RR | 1.26 (0.88-1.80) |
| Bardia et al, 2011(45) | Breast cancer | Women | Regular use | 1581 | 14504 | RR | 0.80 (0.71-0.90) |
|  | Breast cancer | Women | Daily use | 778 | 14504 | RR | 0.71 (0.60-0.83) |
| Bosco et al, 2011(46) | Breast cancer | Women | Regular use | 1275 | 50226 | RR | 0.90 (0.75-1.07) |
|  | Breast cancer | Women | ≥ 5 yrs | 1170 | 50226 | RR | 0.78 (0.58-1.05) |
| Brasky et al, 2014(47) | Breast cancer | Women | Regular use | 3626 | 93272 | HR | 1.11 (1.00-1.24) |
|  | Breast cancer | Women | ≥ 5 yrs | 3213 | 83985 | HR | 1.05 (0.89-1.23) |
| Hollestein et al, 2014(10) | Breast cancer | Women | Regular use | 585 | 55597 | HR | 1.02 (0.97-1.08) |
|  | Breast cancer | Women | Daily use | 585 | 55597 | HR | 1.02 (0.97-1.08) |
|  | Breast cancer | Women | ≥ 5 yrs | 37 | 55597 | HR | 1.18 (0.76-1.82) |
| Kim et al, 2015(48) | Breast cancer | Women | Regular use | 1554 | 50883 | HR | 0.87 (0.68-1.13) |
|  | Breast cancer | Women | Daily use | 1964 | 50883 | HR | 1.07 (0.95-1.21) |
|  | Breast cancer | Women | ≥ 5 yrs | 1771 | 50883 | HR | 0.88 (0.65-1.20) |
| Cao et al, 2016(12) | Breast cancer | Women | Regular use | 607 | 135965 | RR | 0.98 (0.93-1.02) |
| Clarke et al, 2017(49) | Breast cancer | Women | Regular use | 852 | 113479 | HR | 0.84 (0.72-0.98) |
| Yang et al, 2017(50) | Breast cancer | Women | Regular use | 849 | 148739 | HR | 0.81 (0.70-0.92) |
| Bens et al, 2018(51) | Breast cancer | Women | Regular use | 1407 | 62261 | HR | 0.88 (0.72-1.08) |
|  | Breast cancer | Women | Daily use | 1357 | 62261 | HR | 0.97 (0.77-1.23) |
|  | Breast cancer | Women | ≥ 5 yrs | 1313 | 55871 | HR | 0.87 (0.63-1.21) |
| Ajrouche et al, 2019(52) | Breast cancer | Women | Regular use | 2103 | 61087 | SHR | 0.82 (0.54-1.26) |
|  | Breast cancer | Women | ≥ 5 yrs | 2103 | 61087 | SHR | 0.82 (0.54-1.26) |
| Kehm et al, 2019(53) | Breast cancer | Women | Regular use | 2341 | 8233 | HR | 0.63 (0.57-0.71) |
| Tsoi et al, 2019(34) | Breast cancer | Women | Regular use | 4478 | 612509 | RR | 1.14 (1.04-1.25) |
|  | Breast cancer | Women | ≥ 5 yrs | 4478 | 612509 | RR | 1.14 (1.04-1.25) |
| Bertrand et al, 2020(54) | Breast cancer | Women | Regular use | 1594 | 53126 | HR | 0.92 (0.81-1.04) |
|  | Breast cancer | Women | ≥ 5 yrs | 1421 | 53126 | HR | 0.91 (0.78-1.06) |
| Blair et al, 2007(55) | Breast cancer mortality | Women | Post-diagnostic | 48 | 592 | HR | 0.53 (0.30-0.93) |
| Wernli et al, 2011(56) | Breast cancer mortality | Women | Post-diagnostic | 148 | 3058 | HR | 0.64 (0.27-1.37) |
| Barron et al, 2012(57) | Breast cancer mortality | Women | Pre-diagnostic | 361 | 5304 | HR | 0.80 (0.62-1.04) |
|  | Breast cancer mortality | Women | Post-diagnostic | 361 | 5304 | HR | 0.99 (0.68-1.45) |
| Fraser et al, 2014(58) | Breast cancer mortality | Women | Post-diagnostic | 815 | 4627 | HR | 0.42 (0.31-0.55) |
| Holmes et al, 2014(59) | Breast cancer mortality | Women | Post-diagnostic | 165 | 5630 | HR | 0.96 (0.80-1.16) |
| Murray et al, 2014(60) | Breast cancer mortality | Women | Post-diagnostic | 1435 | 7132 | HR | 0.98 (0.81-1.20) |
| Bradley et al, 2016(61) | Breast cancer mortality | Women | Pre-diagnostic | 153 | 2925 | HR | 0.95 (0.68-1.31) |
| Menamin et al, 2017(62) | Breast cancer mortality | Women | Pre-diagnostic | 1215 | 15140 | HR | 0.95 (0.81-1.11) |
|  | Breast cancer mortality | Women | Post-diagnostic | 1190 | 15140 | HR | 0.92 (0.75-1.14) |
| Frisk et al, 2018(63) | Breast cancer mortality | Women | Pre-diagnostic | 1075 | 21140 | HR | 0.93 (0.77-1.12) |
|  | Breast cancer mortality | Women | Post-diagnostic | 1075 | 21201 | HR | 0.99 (0.79-1.23) |
| Wang et al, 2018(64) | Breast cancer mortality | Women | Pre-diagnostic | 226 | 1216 | HR | 0.87 (0.59-1.29) |
| Paganini-Hill et al, 1989(1) | Prostate cancer | Men | Regular use | 131 | 4366 | RR | 0.95 (0.65-1.33) |
|  | Prostate cancer | Men | Daily use | 131 | 4366 | RR | 0.95 (0.65-1.33) |
| Habel et al, 2002(65) | Prostate cancer | Men | Regular use | 2574 | 90100 | RR | 0.76 (0.60-0.98) |
|  | Prostate cancer | Men | Daily use | 2574 | 90100 | RR | 0.76 (0.60-0.98) |
| Ratnasinghe et al, 2004(19) | Prostate cancer | Men | Regular use | 121 | 9869 | RR | 1.11 (0.60-2.05) |
| Platz et al, 2005(66) | Prostate cancer | Men | Regular use | 141 | 1031 | RR | 0.76 (0.54-1.07) |
| Jacobs et al, 2007(6) | Prostate cancer | Men | Regular use | 1986 | 52721 | RR | 1.02 (0.93-1.12) |
|  | Prostate cancer | Men | Daily use | 1986 | 52721 | RR | 1.02 (0.93-1.12) |
|  | Prostate cancer | Men | ≥ 5 yrs | 1301 | 33313 | RR | 0.81 (0.70-0.94) |
| Siemes et al, 2008(7) | Prostate cancer | Men | Regular use | 150 | 4654 | HR | 1.04 (0.73-1.47) |
| Brasky et al, 2010(67) | Prostate cancer | Men | Regular use | 1547 | 34132 | HR | 0.98 (0.87-1.09) |
|  | Prostate cancer | Men | ≥ 5 yrs | 1295 | 34132 | HR | 0.96 (0.83-1.11) |
| Shebl et al, 2012(68) | Prostate cancer | Men | Regular use | 2725 | 22583 | RR | 0.92 (0.85-0.99) |
|  | Prostate cancer | Men | Daily use | 2725 | 22583 | RR | 0.92 (0.85-0.99) |
| Hollestein et al, 2014(10) | Prostate cancer | Men | Regular use | 882 | 53679 | HR | 1.02 (0.98-1.06) |
|  | Prostate cancer | Men | Daily use | 882 | 53679 | HR | 1.02 (0.98-1.06) |
|  | Prostate cancer | Men | ≥ 5 yrs | 93 | 53679 | HR | 1.25 (0.90-1.73) |
| Nordström et al, 2015(69) | Prostate cancer | Men | Regular use | 8430 | 185667 | OR | 1.12 (1.00-1.25) |
| Lapi et al, 2016(70) | Prostate cancer | Men | Regular use | 187 | 13453 | HR | 0.64 (0.48-0.86) |
|  | Prostate cancer | Men | ≥ 5 yrs | 108 | 13453 | HR | 0.42 (0.21-0.91) |
| Skriver et al, 2016(71) | Prostate cancer | Men | Regular use | 34522 | 206742 | OR | 0.94 (0.91-0.97) |
| Downer et al, 2017(72) | Prostate cancer | Men | Regular use | 378 | 22071 | HR | 0.66 (0.50-0.85) |
|  | Prostate cancer | Men | ≥ 5 yrs | 340 | 22071 | HR | 0.66 (0.50-0.86) |
| Ma et al, 2018(73) | Prostate cancer | Men | Regular use | 14870 | 643368 | SIR | 0.87 (0.85-0.88) |
|  | Prostate cancer | Men | ≥ 5 yrs | 6200 | 643368 | SIR | 0.31 (0.30-0.32) |
| Ajrouche et al, 2019(52) | Prostate cancer | Men | Regular use | 2421 | 49938 | SHR | 0.79 (0.71-0.88) |
|  | Prostate cancer | Men | ≥ 5 yrs | 2421 | 49938 | SHR | 0.79 (0.71-0.88) |
| Downer et al, 2019(74) | Prostate cancer | Men | Regular use | 542 | 49409 | HR | 0.80 (0.66-0.96) |
|  | Prostate cancer | Men | ≥ 5 yrs | 59 | 13918 | HR | 0.42 (0.23-0.79) |
|  | Prostate cancer mortality | Men | Post-diagnostic | 804 | 49384 | HR | 0.82 (0.71-0.95) |
| Hurwitz et al, 2019(75) | Prostate cancer | Men | Regular use | 817 | 6594 | HR | 1.05 (0.91-1.22) |
|  | Prostate cancer | Men | Daily use | 463 | 4527 | HR | 1.12 (0.90-1.39) |
|  | Prostate cancer mortality | Men | Post-diagnostic | 90 | 6594 | HR | 0.59 (0.36-0.96) |
| Tsoi et al, 2019(34) | Prostate cancer | Men | Regular use | 6771 | 612509 | RR | 0.95 (0.88-1.03) |
|  | Prostate cancer | Men | ≥ 5 yrs | 6771 | 612509 | RR | 0.95 (0.88-1.03) |
| Prause et al, 2020(76) | Prostate cancer | Men | Regular use | 392 | 4314 | HR | 0.84 (0.62-1.16) |
|  | Prostate cancer | Men | ≥ 5 yrs | 392 | 4314 | HR | 0.84 (0.62-1.16) |
| Skriver et al, 2020(77) | Prostate cancer | Men | Regular use | 1898 | 26339 | HR | 1.02 (0.90-1.15) |
|  | Prostate cancer | Men | Daily use | 1594 | 26339 | HR | 1.06 (0.90-1.25) |
|  | Prostate cancer | Men | ≥ 5 yrs | 1448 | 26339 | HR | 1.02 (0.78-1.33) |
| Choe et al, 2012(78) | Prostate cancer mortality | Men | Post-diagnostic | 334 | 7369 | HR | 0.43 (0.21-0.87) |
| Caon et al, 2014(79) | Prostate cancer mortality | Men | Pre-diagnostic | 1098 | 4419 | HR | 0.91 (0.65-1.28) |
| Flahavan et al, 2014(80) | Prostate cancer mortality | Men | Pre-diagnostic | 276 | 2936 | HR | 0.88 (0.67-1.15) |
| Grytli et al, 2014(81) | Prostate cancer mortality | Men | Post-diagnostic | 1010 | 3561 | HR | 0.94 (0.78-1.14) |
| Jacobs et al, 2014(82) | Prostate cancer mortality | Men | Post-diagnostic | 246 | 7419 | HR | 0.98 (0.74-1.29) |
| Assayag et al, 2015(83) | Prostate cancer mortality | Men | Post-diagnostic | 1793 | 2594 | HR | 1.46 (1.29-1.65) |
| Zhou et al, 2018(84) | Prostate cancer mortality | Men | Post-diagnostic | 244 | 26890 | HR | 0.81 (0.58-1.14) |
|  | Prostate cancer mortality | Men | Pre-diagnostic | 283 | 26890 | HR | 0.98 (0.83-1.17) |
| Paganini-Hill et al, 1989(1) | Lung cancer | General | Regular use | 95 | 11767 | RR | 1.25 (0.71-2.20) |
|  | Lung cancer | General | Daily use | 95 | 11767 | RR | 1.25 (0.71-2.20) |
| Ratnasinghe et al, 2004(19) | Lung cancer | General | Regular use | 410 | 22834 | RR | 0.81 (0.62-1.07) |
| Hayes et al, 2006(85) | Lung cancer | Women | Regular use | 236 | 16644 | HR | 1.10 (0.84-1.43) |
|  | Lung cancer | Women | Daily use | 219 | 13347 | HR | 1.21 (0.92-1.59) |
| Jacobs et al, 2007(6) | Lung cancer | General | Regular use | 656 | 51391 | RR | 1.07 (0.91-1.25) |
|  | Lung cancer | General | Daily use | 656 | 51391 | RR | 1.07 (0.91-1.25) |
|  | Lung cancer | General | ≥ 5 yrs | 439 | 32451 | RR | 0.98 (0.76-1.25) |
| Siemes et al, 2008(7) | Lung cancer | General | Regular use | 84 | 4649 | HR | 0.90 (0.56-1.45) |
| Slatore et al, 2009(86) | Lung cancer | General | Regular use | 569 | 66248 | HR | 0.90 (0.71-1.15) |
| Hollestein et al, 2014(10) | Lung cancer | General | Regular use | 915 | 109276 | HR | 1.06 (1.01-1.10) |
|  | Lung cancer | General | Daily use | 915 | 109276 | HR | 1.06 (1.01-1.10) |
|  | Lung cancer | General | ≥ 5 yrs | 75 | 109276 | HR | 1.17 (0.83-1.64) |
| Baik et al, 2015(87) | Lung cancer | Women | Regular use | 1158 | 143841 | HR | 0.97 (0.84-1.13) |
|  | Lung cancer | Women | ≥ 5 yrs | 1013 | 143841 | HR | 0.95 (0.81-1.12) |
| Cao et al, 2016(12) | Lung cancer | General | Regular use | 2430 | 135965 | RR | 1.05 (0.97-1.14) |
| Tsoi et al, 2019(34) | Lung cancer | General | Regular use | 24908 | 612509 | RR | 0.65 (0.62-0.68) |
|  | Lung cancer | General | ≥ 5 yrs | 24908 | 612509 | RR | 0.65 (0.62-0.68) |
| Kang et al, 2020(88) | Lung cancer | General | Regular use | 5990 | 732199 | HR | 0.99 (0.92-1.06) |
|  | Lung cancer | General | Daily use | NA | NA | HR | 0.95 (0.86-1.06) |

Abbreviations: CRC, colorectal cancer; yrs, years; NA, not available.

**Supplementary Table 3. Basic characteristics and main findings for each RCT.**

| **Author, Year** | **Trial design** | **Outcome** | **Exposure** | **Treatment duration** | **Follow-up period** | **Events** | **Sample size** | **Estimates (95% CI)** |
| --- | --- | --- | --- | --- | --- | --- | --- | --- |
| Peto et al, 1988(89) | Primary + Secondary | CRC | Daily | 6 yrs | 0-12 yrs | 45 | 5139 | 0.82 (0.45-1.50) |
|  | Primary + Secondary | CRC | Daily | 6 yrs | 10-19 yrs | 88 | 5139 | 0.66 (0.43-1.00) |
| Stürmer et al, 1998(3) | Primary + Secondary | CRC | Daily | 5 yrs | 0-12 yrs | 341 | 22071 | 1.03 (0.83-1.27) |
| Rothwell et al, 2010(90) | Primary + Secondary | CRC | Daily | ≥2.5 yrs | ≥20 yrs | 185 | 7383 | 0.69 (0.51, 0.93) |
|  | Primary + Secondary | CRC | Daily | ≥5 yrs | ≥20 yrs | 316 | 14033 | 0.68 (0.54, 0.87) |
| Cook et al, 2013(91) | Primary + Secondary | CRC | Daily | 10.1 yrs | 0-12 yrs | 294 | 39876 | 0.96 (0.76-1.21) |
|  | Primary + Secondary | CRC | Daily | 10.1 yrs | 10-19 yrs | 157 | 39876 | 0.59 (0.42-0.81) |
| Peto et al, 1988(89) | Primary | CRC mortality | Daily | 6 yrs | 0-20 yrs | 99 | 5139 | 0.74 (0.49-1.09) |
| Rothwell et al, 2010(90) | Primary + Secondary | CRC mortality | Daily | ≥2.5 yrs | ≥20 yrs | 119 | 7383 | 0.54 (0.36, 0.80) |
|  | Primary + Secondary | CRC mortality | Daily | ≥5 yrs | ≥20 yrs | 193 | 14033 | 0.57 (0.42, 0.78) |
| Flossmann et al, 2007(92) | Primary | Gastric cancer | Daily | ≥5 yrs | ≥20 yrs | 46 | 6076 | 1.01 (0.54, 1.86) |
| Rothwell et al, 2011(93) | Primary + Secondary | Gastric cancer mortality | Daily | 0-5 yrs | Any | <36 | 23535 | 1.85 (0.81, 4.23) |
|  | Primary + Secondary | Gastric cancer mortality | Daily | ≥5 yrs | Any | <36 | 23535 | 3.09 (0.64, 14.91) |
|  | Primary + Secondary | Gastric cancer mortality | Daily | ≥5 yrs | 0-10 yrs | <71 | 10502 | 1.36 (0.64, 2.90) |
|  | Primary + Secondary | Gastric cancer mortality | Daily | ≥5 yrs | 10-20 yrs | <71 | 10502 | 0.42 (0.23, 0.79) |
|  | Primary + Secondary | Gastric cancer mortality | Daily | ≥5 yrs | 0-20 yrs | 71 | 10502 | 0.69 (0.43, 1.10) |
| Flossmann et al, 2007(92) | Primary | Breast cancer | Daily | ≥5 yrs | ≥20 yrs | 12 | 6076 | 0.90 (0.26, 3.07) |
|  | Primary | Prostate cancer | Daily | ≥5 yrs | ≥20 yrs | 313 | 6076 | 0.87 (0.69, 1.10) |
| Rothwell et al, 2011(93) | Primary + Secondary | Prostate cancer mortality | Daily | 0-5 yrs | Any | <37 | 23535 | 0.70 (0.29, 1.73) |
|  | Primary + Secondary | Prostate cancer mortality | Daily | ≥5 yrs | Any | <37 | 23535 | 0.52 (0.20, 1.34) |
|  | Primary + Secondary | Prostate cancer mortality | Daily | ≥5 yrs | 0-10 yrs | <210 | 10502 | 0.83 (0.47, 1.46) |
|  | Primary + Secondary | Prostate cancer mortality | Daily | ≥5 yrs | 10-20 yrs | <210 | 10502 | 0.80 (0.58, 1.09) |
|  | Primary + Secondary | Prostate cancer mortality | Daily | ≥5 yrs | 0-20 yrs | 210 | 10502 | 0.81 (0.61, 1.06) |
| Peto et al, 1988(89) | Primary | Lung cancer | Daily | 6 yrs | Any | 28 | 5199 | 0.64 (0.29-1.41) |
| Lee et al, 1995(94) | Primary | Lung cancer | Any | NA | Any | 128 | 22071 | 0.88 (0.62-1.25) |
| Flossmann et al, 2007(92) | Primary | Lung cancer | Daily | ≥5 yrs | ≥20 yrs | 169 | 6076 | 0.96 (0.70, 1.32) |
| Cook et al, 2013(91) | Primary | Lung cancer | Daily | 10.1 yrs | Any | 431 | 39876 | 1.04 (0.86-1.26) |
| Rothwell et al, 2011(93) | Primary + Secondary | Lung cancer mortality | Daily | 0-5 yrs | Any | <198 | 23535 | 0.92 (0.65, 1.30) |
|  | Primary + Secondary | Lung cancer mortality | Daily | ≥5 yrs | Any | <198 | 23535 | 0.68 (0.42, 1.10) |
|  | Primary + Secondary | Lung cancer mortality | Daily | ≥5 yrs | 0-10 yrs | <326 | 10502 | 0.68 (0.50, 0.92) |
|  | Primary + Secondary | Lung cancer mortality | Daily | ≥5 yrs | 10-20 yrs | <326 | 10502 | 0.75 (0.55, 1.02) |
|  | Primary + Secondary | Lung cancer mortality | Daily | ≥5 yrs | 0-20 yrs | 326 | 10502 | 0.71 (0.58, 0.89) |

Abbreviations: CRC, colorectal cancer; yrs, years; NA, not available.

**Reference**

1. Paganini-Hill A, Chao A, Ross RK, Henderson BE. Aspirin use and chronic diseases: a cohort study of the elderly. *BMJ* (1989) 299(6710):1247-50. doi: 10.1136/bmj.299.6710.1247

2. Giovannucci E, Rimm EB, Stampfer MJ, Colditz GA, Ascherio A, Willett WC. Aspirin use and the risk for colorectal cancer and adenoma in male health professionals. *Ann Intern Med* (1994) 121(4):241-6. doi: 10.7326/0003-4819-121-4-199408150-00001

3. Sturmer T, Glynn RJ, Lee IM, Manson JE, Buring JE, Hennekens CH. Aspirin use and colorectal cancer: post-trial follow-up data from the Physicians' Health Study. *Ann Intern Med* (1998) 128(9):713-20. doi: 10.7326/0003-4819-128-9-199805010-00003

4. Larsson SC, Giovannucci E, Wolk A. Long-term aspirin use and colorectal cancer risk: a cohort study in Sweden. *Br J Cancer* (2006) 95(9):1277-9. doi: 10.1038/sj.bjc.6603442

5. Mahipal A, Anderson KE, Limburg PJ, Folsom AR. Nonsteroidal anti-inflammatory drugs and subsite-specific colorectal cancer incidence in the Iowa women's health study. *Cancer Epidemiol Biomarkers Prev* (2006) 15(10):1785-90. doi: 10.1158/1055-9965.EPI-05-0674

6. Jacobs EJ, Thun MJ, Bain EB, Rodriguez C, Henley SJ, Calle EE. A large cohort study of long-term daily use of adult-strength aspirin and cancer incidence. *J Natl Cancer Inst* (2007) 99(8):608-15. doi: 10.1093/jnci/djk132

7. Siemes C, Visser LE, Coebergh JW, Hofman A, Uitterlinden AG, Stricker BH. Protective effect of NSAIDs on cancer and influence of COX-2 C(-765G) genotype. *Curr Cancer Drug Targets* (2008) 8(8):753-64. doi: 10.2174/156800908786733414

8. Friis S, Poulsen AH, Sorensen HT, Tjonneland A, Overvad K, Vogel U, et al. Aspirin and other non-steroidal anti-inflammatory drugs and risk of colorectal cancer: a Danish cohort study. *Cancer Causes Control* (2009) 20(5):731-40. doi: 10.1007/s10552-008-9286-7

9. Ruder EH, Laiyemo AO, Graubard BI, Hollenbeck AR, Schatzkin A, Cross AJ. Non-steroidal anti-inflammatory drugs and colorectal cancer risk in a large, prospective cohort. *Am J Gastroenterol* (2011) 106(7):1340-50. doi: 10.1038/ajg.2011.38

10. Hollestein LM, van Herk-Sukel MP, Ruiter R, de Vries E, Mathijssen RH, Wiemer EA, et al. Incident cancer risk after the start of aspirin use: results from a Dutch population-based cohort study of low dose aspirin users. *Int J Cancer* (2014) 135(1):157-65. doi: 10.1002/ijc.28634

11. Lin CC, Lai MS, Shau WY. Can aspirin reduce the risk of colorectal cancer in people with diabetes? A population-based cohort study. *Diabet Med* (2015) 32(3):324-31. doi: 10.1111/dme.12596

12. Cao Y, Nishihara R, Wu K, Wang M, Ogino S, Willett WC, et al. Population-wide Impact of Long-term Use of Aspirin and the Risk for Cancer. *JAMA Oncol* (2016) 2(6):762-9. doi: 10.1001/jamaoncol.2015.6396

13. Park SY, Wilkens LR, Kolonel LN, Monroe KR, Haiman CA, Marchand LL. Exploring Differences in the Aspirin-Colorectal Cancer Association by Sex and Race/Ethnicity: The Multiethnic Cohort Study. *Cancer Epidemiol Biomarkers Prev* (2017) 26(2):162-9. doi: 10.1158/1055-9965.EPI-16-0560

14. Kuo CN, Pan JJ, Huang YW, Tsai HJ, Chang WC. Association between Nonsteroidal Anti-Inflammatory Drugs and Colorectal Cancer: A Population-Based Case-Control Study. *Cancer Epidemiol Biomarkers Prev* (2018) 27(7):737-45. doi: 10.1158/1055-9965.EPI-17-0876

15. Tsoi KK, Chan FC, Hirai HW, Sung JJ. Risk of gastrointestinal bleeding and benefit from colorectal cancer reduction from long-term use of low-dose aspirin: A retrospective study of 612 509 patients. *J Gastroenterol Hepatol* (2018) 33(10):1728-36. doi: 10.1111/jgh.14261

16. Cea Soriano L, Vora P, Soriano-Gabarro M, Garcia Rodriguez LA. The effect of low-dose aspirin on colorectal cancer prevention and gastrointestinal bleeding according to bodyweight and body mass index: Analysis of UK primary care data. *Int J Cardiol* (2019) 297:135-9. doi: 10.1016/j.ijcard.2019.08.001

17. Rodriguez-Miguel A, Garcia-Rodriguez LA, Gil M, Montoya H, Rodriguez-Martin S, de Abajo FJ. Clopidogrel and Low-Dose Aspirin, Alone or Together, Reduce Risk of Colorectal Cancer. *Clin Gastroenterol Hepatol* (2019) 17(10):2024-33 e2. doi: 10.1016/j.cgh.2018.12.012

18. Troelsen FS, Farkas DK, Erichsen R, Sorensen HT. Risk of lower gastrointestinal bleeding and colorectal neoplasms following initiation of low-dose aspirin: a Danish population-based cohort study. *BMJ Open Gastroenterol* (2020) 7(1). doi: 10.1136/bmjgast-2020-000453

19. Ratnasinghe LD, Graubard BI, Kahle L, Tangrea JA, Taylor PR, Hawk E. Aspirin use and mortality from cancer in a prospective cohort study. *Anticancer Res* (2004) 24(5B):3177-84.

20. Chan AT, Ogino S, Fuchs CS. Aspirin use and survival after diagnosis of colorectal cancer. *JAMA* (2009) 302(6):649-58. doi: 10.1001/jama.2009.1112

21. Coghill AE, Newcomb PA, Campbell PT, Burnett-Hartman AN, Adams SV, Poole EM, et al. Prediagnostic non-steroidal anti-inflammatory drug use and survival after diagnosis of colorectal cancer. *Gut* (2011) 60(4):491-8. doi: 10.1136/gut.2010.221143

22. McCowan C, Munro AJ, Donnan PT, Steele RJ. Use of aspirin post-diagnosis in a cohort of patients with colorectal cancer and its association with all-cause and colorectal cancer specific mortality. *Eur J Cancer* (2013) 49(5):1049-57. doi: 10.1016/j.ejca.2012.10.024

23. Cardwell CR, Kunzmann AT, Cantwell MM, Hughes C, Baron JA, Powe DG, et al. Low-dose aspirin use after diagnosis of colorectal cancer does not increase survival: a case-control analysis of a population-based cohort. *Gastroenterology* (2014) 146(3):700-8 e2. doi: 10.1053/j.gastro.2013.11.005

24. Goh CH, Leong WQ, Chew MH, Pan YS, Tony LK, Chew L, et al. Post-operative aspirin use and colorectal cancer-specific survival in patients with stage I-III colorectal cancer. *Anticancer Res* (2014) 34(12):7407-14.

25. Bains SJ, Mahic M, Myklebust TA, Smastuen MC, Yaqub S, Dorum LM, et al. Aspirin As Secondary Prevention in Patients With Colorectal Cancer: An Unselected Population-Based Study. *J Clin Oncol* (2016) 34(21):2501-8. doi: 10.1200/JCO.2015.65.3519

26. Gray RT, Coleman HG, Hughes C, Murray LJ, Cardwell CR. Low-dose aspirin use and survival in colorectal cancer: results from a population-based cohort study. *BMC Cancer* (2018) 18(1):228. doi: 10.1186/s12885-018-4142-y

27. Ventura L, Miccinesi G, Barchielli A, Manneschi G, Puliti D, Mantellini P, et al. Does low-dose aspirin use for cardiovascular disease prevention reduce colorectal cancer deaths? A comparison of two cohorts in the Florence district, Italy. *Eur J Cancer Prev* (2018) 27(2):134-9. doi: 10.1097/CEJ.0000000000000319

28. Loomans-Kropp HA, Pinsky P, Cao Y, Chan AT, Umar A. Association of Aspirin Use With Mortality Risk Among Older Adult Participants in the Prostate, Lung, Colorectal, and Ovarian Cancer Screening Trial. *JAMA Netw Open* (2019) 2(12):e1916729. doi: 10.1001/jamanetworkopen.2019.16729

29. Thun MJ, Namboodiri MM, Calle EE, Flanders WD, Heath CW, Jr. Aspirin use and risk of fatal cancer. *Cancer Res* (1993) 53(6):1322-7.

30. Abnet CC, Freedman ND, Kamangar F, Leitzmann MF, Hollenbeck AR, Schatzkin A. Non-steroidal anti-inflammatory drugs and risk of gastric and oesophageal adenocarcinomas: results from a cohort study and a meta-analysis. *Br J Cancer* (2009) 100(3):551-7. doi: 10.1038/sj.bjc.6604880

31. Epplein M, Nomura AM, Wilkens LR, Henderson BE, Kolonel LN. Nonsteroidal antiinflammatory drugs and risk of gastric adenocarcinoma: the multiethnic cohort study. *Am J Epidemiol* (2009) 170(4):507-14. doi: 10.1093/aje/kwp162

32. Spence AD, Busby J, Johnston BT, Baron JA, Hughes CM, Coleman HG, et al. Low-Dose Aspirin Use Does Not Increase Survival in 2 Independent Population-Based Cohorts of Patients With Esophageal or Gastric Cancer. *Gastroenterology* (2018) 154(4):849-60. doi: 10.1053/j.gastro.2017.10.044

33. Kim MH, Chang J, Kim WJ, Banerjee S, Park SM. Cumulative Dose Threshold for the Chemopreventive Effect of Aspirin Against Gastric Cancer. *Am J Gastroenterol* (2018) 113(6):845-54. doi: 10.1038/s41395-018-0097-5

34. Tsoi KKF, Ho JMW, Chan FCH, Sung JJY. Long-term use of low-dose aspirin for cancer prevention: A 10-year population cohort study in Hong Kong. *Int J Cancer* (2019) 145(1):267-73. doi: 10.1002/ijc.32083

35. Garcia Rodriguez LA, Soriano-Gabarro M, Vora P, Cea Soriano L. Low-dose aspirin and risk of gastric and oesophageal cancer: A population-based study in the United Kingdom using The Health Improvement Network. *Int J Cancer* (2020) 147(9):2394-404. doi: 10.1002/ijc.33022

36. Friedman GD, Ury HK. Initial screening for carcinogenicity of commonly used drugs. *J Natl Cancer Inst* (1980) 65(4):723-33. doi: 10.1093/jnci/65.4.723

37. Harris RE, Kasbari S, Farrar WB. Prospective study of nonsteroidal anti-inflammatory drugs and breast cancer. *Oncol Rep* (1999) 6(1):71-3.

38. Marshall SF, Bernstein L, Anton-Culver H, Deapen D, Horn-Ross PL, Mohrenweiser H, et al. Nonsteroidal anti-inflammatory drug use and breast cancer risk by stage and hormone receptor status. *J Natl Cancer Inst* (2005) 97(11):805-12. doi: 10.1093/jnci/dji140

39. Gallicchio L, Visvanathan K, Burke A, Hoffman SC, Helzlsouer KJ. Nonsteroidal anti-inflammatory drugs and the risk of developing breast cancer in a population-based prospective cohort study in Washington County, MD. *Int J Cancer* (2007) 121(1):211-15. doi: 10.1002/ijc.22656

40. Gill JK, Maskarinec G, Wilkens LR, Pike MC, Henderson BE, Kolonel LN. Nonsteroidal antiinflammatory drugs and breast cancer risk: the multiethnic cohort. *Am J Epidemiol* (2007) 166(10):1150-8. doi: 10.1093/aje/kwm195

41. Friis S, Thomassen L, Sorensen HT, Tjonneland A, Overvad K, Cronin-Fenton DP, et al. Nonsteroidal anti-inflammatory drug use and breast cancer risk: a Danish cohort study. *Eur J Cancer Prev* (2008) 17(2):88-96. doi: 10.1097/CEJ.0b013e3282b6fd55

42. Gierach GL, Lacey JV, Jr., Schatzkin A, Leitzmann MF, Richesson D, Hollenbeck AR, et al. Nonsteroidal anti-inflammatory drugs and breast cancer risk in the National Institutes of Health-AARP Diet and Health Study. *Breast Cancer Res* (2008) 10(2):R38. doi: 10.1186/bcr2089

43. Ready A, Velicer CM, McTiernan A, White E. NSAID use and breast cancer risk in the VITAL cohort. *Breast Cancer Res Treat* (2008) 109(3):533-43. doi: 10.1007/s10549-007-9665-x

44. Eliassen AH, Chen WY, Spiegelman D, Willett WC, Hunter DJ, Hankinson SE. Use of aspirin, other nonsteroidal anti-inflammatory drugs, and acetaminophen and risk of breast cancer among premenopausal women in the Nurses' Health Study II. *Arch Intern Med* (2009) 169(2):115-21; discussion 21. doi: 10.1001/archinternmed.2008.537

45. Bardia A, Olson JE, Vachon CM, Lazovich D, Vierkant RA, Wang AH, et al. Effect of aspirin and other NSAIDs on postmenopausal breast cancer incidence by hormone receptor status: results from a prospective cohort study. *Breast Cancer Res Treat* (2011) 126(1):149-55. doi: 10.1007/s10549-010-1074-x

46. Bosco JL, Palmer JR, Boggs DA, Hatch EE, Rosenberg L. Regular aspirin use and breast cancer risk in US Black women. *Cancer Causes Control* (2011) 22(11):1553-61. doi: 10.1007/s10552-011-9832-6

47. Brasky TM, Liu J, White E, Peters U, Potter JD, Walter RB, et al. Non-steroidal anti-inflammatory drugs and cancer risk in women: results from the Women's Health Initiative. *Int J Cancer* (2014) 135(8):1869-83. doi: 10.1002/ijc.28823

48. Kim S, Shore DL, Wilson LE, Sanniez EI, Kim JH, Taylor JA, et al. Lifetime use of nonsteroidal anti-inflammatory drugs and breast cancer risk: results from a prospective study of women with a sister with breast cancer. *BMC Cancer* (2015) 15:960. doi: 10.1186/s12885-015-1979-1

49. Clarke CA, Canchola AJ, Moy LM, Neuhausen SL, Chung NT, Lacey JV, Jr., et al. Regular and low-dose aspirin, other non-steroidal anti-inflammatory medications and prospective risk of HER2-defined breast cancer: the California Teachers Study. *Breast Cancer Res* (2017) 19(1):52. doi: 10.1186/s13058-017-0840-7

50. Yang YS, Kornelius E, Chiou JY, Lai YR, Lo SC, Peng CH, et al. Low-Dose Aspirin Reduces Breast Cancer Risk in Women with Diabetes: A Nationwide Retrospective Cohort Study in Taiwan. *J Womens Health (Larchmt)* (2017) 26(12):1278-84. doi: 10.1089/jwh.2016.6040

51. Bens A, Friis S, Dehlendorff C, Jensen MB, Ejlertsen B, Kroman N, et al. Low-dose aspirin use and risk of contralateral breast cancer: a Danish nationwide cohort study. *Prev Med* (2018) 116:186-93. doi: 10.1016/j.ypmed.2018.09.015

52. Ajrouche A, De Rycke Y, Dalichampt M, Messika Zeitoun D, Hulot JS, Estellat C, et al. Reduced risk of cancer among low-dose aspirin users: Data from French health care databases. *Pharmacoepidemiol Drug Saf* (2019) 28(9):1258-66. doi: 10.1002/pds.4870

53. Kehm RD, Hopper JL, John EM, Phillips KA, MacInnis RJ, Dite GS, et al. Regular use of aspirin and other non-steroidal anti-inflammatory drugs and breast cancer risk for women at familial or genetic risk: a cohort study. *Breast Cancer Res* (2019) 21(1):52. doi: 10.1186/s13058-019-1135-y

54. Bertrand KA, Bethea TN, Gerlovin H, Coogan PF, Barber L, Rosenberg L, et al. Aspirin use and risk of breast cancer in African American women. *Breast Cancer Res* (2020) 22(1):96. doi: 10.1186/s13058-020-01335-1

55. Blair CK, Sweeney C, Anderson KE, Folsom AR. NSAID use and survival after breast cancer diagnosis in post-menopausal women. *Breast Cancer Res Treat* (2007) 101(2):191-7. doi: 10.1007/s10549-006-9277-x

56. Wernli KJ, Hampton JM, Trentham-Dietz A, Newcomb PA. Use of antidepressants and NSAIDs in relation to mortality in long-term breast cancer survivors. *Pharmacoepidemiol Drug Saf* (2011) 20(2):131-7. doi: 10.1002/pds.2064

57. Barron TI, Flahavan EM, Sharp L, Bennett K, Visvanathan K. Recent prediagnostic aspirin use, lymph node involvement, and 5-year mortality in women with stage I-III breast cancer: a nationwide population-based cohort study. *Cancer Res* (2014) 74(15):4065-77. doi: 10.1158/0008-5472.CAN-13-2679

58. Fraser DM, Sullivan FM, Thompson AM, McCowan C. Aspirin use and survival after the diagnosis of breast cancer: a population-based cohort study. *Br J Cancer* (2014) 111(3):623-7. doi: 10.1038/bjc.2014.264

59. Holmes MD, Olsson H, Pawitan Y, Holm J, Lundholm C, Andersson TM, et al. Aspirin intake and breast cancer survival - a nation-wide study using prospectively recorded data in Sweden. *BMC Cancer* (2014) 14:391. doi: 10.1186/1471-2407-14-391

60. Murray LJ, Cooper JA, Hughes CM, Powe DG, Cardwell CR. Post-diagnostic prescriptions for low-dose aspirin and breast cancer-specific survival: a nested case-control study in a breast cancer cohort from the UK Clinical Practice Research Datalink. *Breast Cancer Res* (2014) 16(2):R34. doi: 10.1186/bcr3638

61. Bradley MC, Black A, Freedman AN, Barron TI. Prediagnostic aspirin use and mortality in women with stage I to III breast cancer: A cohort study in the Prostate, Lung, Colorectal, and Ovarian Cancer Screening Trial. *Cancer* (2016) 122(13):2067-75. doi: 10.1002/cncr.30004

62. Mc Menamin UC, Cardwell CR, Hughes CM, Murray LJ. Low-dose aspirin use and survival in breast cancer patients: A nationwide cohort study. *Cancer Epidemiol* (2017) 47:20-7. doi: 10.1016/j.canep.2016.12.008

63. Frisk G, Ekberg S, Lidbrink E, Eloranta S, Sund M, Fredriksson I, et al. No association between low-dose aspirin use and breast cancer outcomes overall: a Swedish population-based study. *Breast Cancer Res* (2018) 20(1):142. doi: 10.1186/s13058-018-1065-0

64. Wang T, Parada H, McClain KM, Bradshaw PT, Terry MB, Teitelbaum SL, et al. Pre-diagnostic aspirin use and mortality after breast cancer. *Cancer Causes Control* (2018) 29(4-5):417-25. doi: 10.1007/s10552-018-1020-5

65. Habel LA, Zhao W, Stanford JL. Daily aspirin use and prostate cancer risk in a large, multiracial cohort in the US. *Cancer Causes Control* (2002) 13(5):427-34. doi: 10.1023/a:1015788502099

66. Platz EA, Rohrmann S, Pearson JD, Corrada MM, Watson DJ, De Marzo AM, et al. Nonsteroidal anti-inflammatory drugs and risk of prostate cancer in the Baltimore Longitudinal Study of Aging. *Cancer Epidemiol Biomarkers Prev* (2005) 14(2):390-6. doi: 10.1158/1055-9965

67. Brasky TM, Velicer CM, Kristal AR, Peters U, Potter JD, White E. Nonsteroidal anti-inflammatory drugs and prostate cancer risk in the VITamins And Lifestyle (VITAL) cohort. *Cancer Epidemiol Biomarkers Prev* (2010) 19(12):3185-8. doi: 10.1158/1055-9965.EPI-10-0942

68. Shebl FM, Sakoda LC, Black A, Koshiol J, Andriole GL, Grubb R, et al. Aspirin but not ibuprofen use is associated with reduced risk of prostate cancer: a PLCO study. *Br J Cancer* (2012) 107(1):207-14. doi: 10.1038/bjc.2012.227

69. Nordstrom T, Clements M, Karlsson R, Adolfsson J, Gronberg H. The risk of prostate cancer for men on aspirin, statin or antidiabetic medications. *Eur J Cancer* (2015) 51(6):725-33. doi: 10.1016/j.ejca.2015.02.003

70. Lapi F, Levi M, Simonetti M, Cancian M, Parretti D, Cricelli I, et al. Risk of prostate cancer in low-dose aspirin users: A retrospective cohort study. *Int J Cancer* (2016) 139(1):205-11. doi: 10.1002/ijc.30061

71. Skriver C, Dehlendorff C, Borre M, Brasso K, Sorensen HT, Hallas J, et al. Low-dose aspirin or other nonsteroidal anti-inflammatory drug use and prostate cancer risk: a nationwide study. *Cancer Causes Control* (2016) 27(9):1067-79. doi: 10.1007/s10552-016-0785-7

72. Downer MK, Allard CB, Preston MA, Gaziano JM, Stampfer MJ, Mucci LA, et al. Regular Aspirin Use and the Risk of Lethal Prostate Cancer in the Physicians' Health Study. *Eur Urol* (2017) 72(5):821-7. doi: 10.1016/j.eururo.2017.01.044

73. Ma Y, Brusselaers N. Maintenance use of aspirin or other non-steroidal anti-inflammatory drugs (NSAIDs) and prostate cancer risk. *Prostate Cancer Prostatic Dis* (2018) 21(1):147-52. doi: 10.1038/s41391-017-0021-x

74. Downer MK, Allard CB, Preston MA, Wilson KM, Kenfield SA, Chan JM, et al. Aspirin Use and Lethal Prostate Cancer in the Health Professionals Follow-up Study. *Eur Urol Oncol* (2019) 2(2):126-34. doi: 10.1016/j.euo.2018.07.002

75. Hurwitz LM, Joshu CE, Barber JR, Prizment AE, Vitolins MZ, Jones MR, et al. Aspirin and Non-Aspirin NSAID Use and Prostate Cancer Incidence, Mortality, and Case Fatality in the Atherosclerosis Risk in Communities Study. *Cancer Epidemiol Biomarkers Prev* (2019) 28(3):563-9. doi: 10.1158/1055-9965.EPI-18-0965

76. Prause LW, Manka L, Millan C, Lang E, Wyler SF, Grobholz R, et al. Influence of regular aspirin intake on PSA values, prostate cancer incidence and overall survival in a prospective screening trial (ERSPC Aarau). *World J Urol* (2020) 38(10):2485-91. doi: 10.1007/s00345-019-03054-5

77. Skriver C, Dehlendorff C, Borre M, Brasso K, Larsen SB, Tjonneland A, et al. Associations of low-dose aspirin or other NSAID use with prostate cancer risk in the Danish Diet, Cancer and Health Study. *Cancer Causes Control* (2020) 31(2):139-51. doi: 10.1007/s10552-019-01252-5

78. Choe KS, Cowan JE, Chan JM, Carroll PR, D'Amico AV, Liauw SL. Aspirin use and the risk of prostate cancer mortality in men treated with prostatectomy or radiotherapy. *J Clin Oncol* (2012) 30(28):3540-4. doi: 10.1200/JCO.2011.41.0308

79. Caon J, Paquette M, Hamm J, Pickles T. Does Statin or ASA Affect Survival When Prostate Cancer Is Treated with External Beam Radiation Therapy? *Prostate Cancer* (2014) 2014:184297. doi: 10.1155/2014/184297

80. Flahavan EM, Bennett K, Sharp L, Barron TI. A cohort study investigating aspirin use and survival in men with prostate cancer. *Ann Oncol* (2014) 25(1):154-9. doi: 10.1093/annonc/mdt428

81. Grytli HH, Fagerland MW, Fossa SD, Tasken KA. Association between use of beta-blockers and prostate cancer-specific survival: a cohort study of 3561 prostate cancer patients with high-risk or metastatic disease. *Eur Urol* (2014) 65(3):635-41. doi: 10.1016/j.eururo.2013.01.007

82. Jacobs CD, Chun SG, Yan J, Xie XJ, Pistenmaa DA, Hannan R, et al. Aspirin improves outcome in high risk prostate cancer patients treated with radiation therapy. *Cancer Biol Ther* (2014) 15(6):699-706. doi: 10.4161/cbt.28554

83. Assayag J, Pollak MN, Azoulay L. The use of aspirin and the risk of mortality in patients with prostate cancer. *J Urol* (2015) 193(4):1220-5. doi: 10.1016/j.juro.2014.11.018

84. Zhou CK, Daugherty SE, Liao LM, Freedman ND, Abnet CC, Pfeiffer R, et al. Do Aspirin and Other NSAIDs Confer a Survival Benefit in Men Diagnosed with Prostate Cancer? A Pooled Analysis of NIH-AARP and PLCO Cohorts. *Cancer Prev Res (Phila)* (2017) 10(7):410-20. doi: 10.1158/1940-6207.CAPR-17-0033

85. Hayes JH, Anderson KE, Folsom AR. Association between nonsteroidal anti-inflammatory drug use and the incidence of lung cancer in the Iowa women's health study. *Cancer Epidemiol Biomarkers Prev* (2006) 15(11):2226-31. doi: 10.1158/1055-9965.EPI-06-0172

86. Slatore CG, Au DH, Littman AJ, Satia JA, White E. Association of nonsteroidal anti-inflammatory drugs with lung cancer: results from a large cohort study. *Cancer Epidemiol Biomarkers Prev* (2009) 18(4):1203-7. doi: 10.1158/1055-9965.EPI-08-1110

87. Baik CS, Brasky TM, Pettinger M, Luo J, Gong Z, Wactawski-Wende J, et al. Nonsteroidal Anti-Inflammatory Drug and Aspirin Use in Relation to Lung Cancer Risk among Postmenopausal Women. *Cancer Epidemiol Biomarkers Prev* (2015) 24(5):790-7. doi: 10.1158/1055-9965.EPI-14-1322

88. Kang J, Jeong SM, Shin DW, Cho M, Cho JH, Kim J. The Associations of Aspirin, Statins, and Metformin With Lung Cancer Risk and Related Mortality: A Time-Dependent Analysis of Population-Based Nationally Representative Data. *J Thorac Oncol* (2021) 16(1):76-88. doi: 10.1016/j.jtho.2020.08.021

89. Peto R, Gray R, Collins R, Wheatley K, Hennekens C, Jamrozik K, et al. Randomised trial of prophylactic daily aspirin in British male doctors. *Br Med J (Clin Res Ed)* (1988) 296(6618):313-6. doi: 10.1136/bmj.296.6618.313

90. Rothwell PM, Wilson M, Elwin CE, Norrving B, Algra A, Warlow CP, et al. Long-term effect of aspirin on colorectal cancer incidence and mortality: 20-year follow-up of five randomised trials. *Lancet* (2010) 376(9754):1741-50. doi: 10.1016/S0140-6736(10)61543-7

91. Cook NR, Lee IM, Zhang SM, Moorthy MV, Buring JE. Alternate-day, low-dose aspirin and cancer risk: long-term observational follow-up of a randomized trial. *Ann Intern Med* (2013) 159(2):77-85. doi: 10.7326/0003-4819-159-2-201307160-00002

92. Flossmann E, Rothwell PM, British Doctors Aspirin T, the UKTIAAT. Effect of aspirin on long-term risk of colorectal cancer: consistent evidence from randomised and observational studies. *Lancet* (2007) 369(9573):1603-13. doi: 10.1016/S0140-6736(07)60747-8

93. Rothwell PM, Fowkes FG, Belch JF, Ogawa H, Warlow CP, Meade TW. Effect of daily aspirin on long-term risk of death due to cancer: analysis of individual patient data from randomised trials. *Lancet* (2011) 377(9759):31-41. doi: 10.1016/S0140-6736(10)62110-1

94. Lee IM, Manson JE, Hennekens CH, Buring JE. Low-dose aspirin and risk of cancer-the Physicians Health Study [abstract]. *Am J Epidemiol* (1995) 141: S28–S28.
